# Effect of Fluorophilic- and Hydrophobic-Modified Polyglycerol-Based Coatings on the Wettability of Low Surface Energy Polymers

**DOI:** 10.1021/acs.langmuir.4c04220

**Published:** 2025-01-27

**Authors:** Florian Junge, Rainer Haag

**Affiliations:** Institut für Chemie und Biochemie, Freie Universität Berlin, Takustrasse 3, 14195 Berlin, Germany

## Abstract

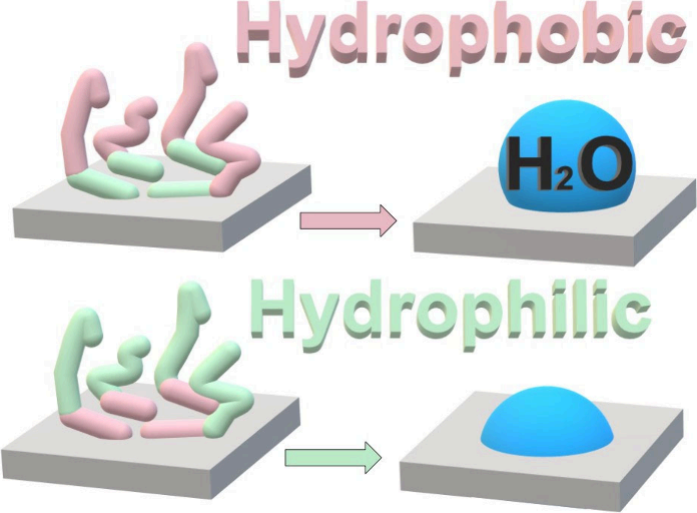

Catechol-derived polymers form stable coatings on a wide range of materials including challenging to coat low surface energy polymers. Whether modification of the coating polymer with fluorophilic or hydrophobic groups is a successful approach to further favor the coating of hydrophobic or fluorophilic surfaces with catechol-based polymers remains ambiguous. Herein, we report the effect of a series of catechol-derived polyglycerol (PG)-based coatings and monolayer coatings on the wettability of polytetrafluoroethylene (PTFE), polystyrene, and poly(methyl methacrylate) surfaces. Coatings with a longer hydrophilic PG block resulted in surface coatings with water contact angles (WCAs) around 60° independently of the modification and substrate, while coatings with a longer hydrophobic anchoring block possessed more diverse WCAs up to (129 ± 10)°. Despite the generally small impact of the fluorophilic modification for most substrate/coating combinations, some fluorophilic modified coatings reduce the WCA of PTFE below Berg’s limit of 65°, indicating a shielding of fluorous segments from the surface.

## Introduction

Many common polymeric biomaterials and plastics in laboratory and outdoor applications suffer from biofouling induced by the initial adsorption of protein. Changing a polymer’s surface energy by hydrophilic, zwitterionic, or superhydrophobic coatings is a common strategy to boost the surface’s resistance to adsorption of proteins.^1−4^ Specifically polyglycerol, a biocompatible polyether that is more hydrophilic than the structurally related polyethylene glycol,^5^ proved to be a versatile, easily functionalizable polymer for the creation of superhydrophilic to superhydrophobic (after fluoroalkylation or micropatterning) coatings using biomimetic catechol-cross-linking chemistry.^6−10^ Already weakly basic conditions (pH approximately 8.5) are sufficient to initiate an oxidative cross-linking of catechol units while forming an insoluble coating layer.^11−13^ Besides the possibility of oxidative cross-linking, catechol units provide a rare combination of aromaticity, moderate hydrophobicity, and two hydroxyl groups in *ortho*-position.^14^ Consequently, catechol-derived units adsorb and bind not only on polar high surface energy substrates, like metal oxides through complexation or silica through hydrogen bonding, but also on low surface energy substrates with no polar groups, like polystyrene (PS) through π–π stacking or polytetrafluoroethylene (PTFE) through hydrophobic interactions.^14−16^ However, the hydrophobicity of catechol-derived groups is often not sufficient to provide effective binding to hydrophobic surfaces. For this purpose, combination with more hydrophobic groups is advantageous; for example, the presence of hydrophobic tryptophan amino acids in the structure of 3,4-dihydroxyphenyl alanine (DOPA)-based proteins increased the adsorption of the whole coating polymer to a hydrophobic self-assembled monolayer substrate.^15^ Yu et al.^17^ reported a linear polyglycerol (lPG) block copolymer which was equipped with hydrophobic phenyl groups (besides catechol groups) to promote adhesion on hydrophobic polymers including PS and PTFE. Many other scientists further showcased the suitability of catechol-^18^ and similar dopamine-derived structures for the coating and dispersion of PTFE, e.g., for the coating of expanded PTFE as implants for the treatment of congenital diaphragmatic hernia,^19^ for the coating of PTFE membranes,^20,21^ for the coating of PTFE micropowders for enhanced dispersion in aqueous lubricants,^22^ or for the coating of microfluidic PTFE chips.^23^

Fluorophilic interactions have been observed between per- and polyfluorinated molecules in many fields of chemistry and typically lead to the separation of fluorinated species in an exclusive phase.^24^ Despite the surprisingly good coating of PTFE by catechol-derived structures, whether fluorophilic modification of the latter may lead to an even better attraction between the coating and the fluoropolymer surface remains obscure. Baby et al.^25^ reported an increased bond strength of epoxy adhesive joints of PTFE substrates after pretreating the PTFE substrate with a tetrafluorobutyl-modified epoxy-dopamine prepolymer, suggesting a beneficial contribution of fluorophilic interactions in the bonding process. Interestingly, the tetrafluorobutyl-modified prepolymer also turned out to be the strongest adhesion promoter for (nonfluorinated) high-density polyethylene substrates. Also, density functional theory calculations showed attractive C–F---O and F---H–O but no F---F interactions between the prepolymer and the substrate. A couple of years earlier, hydrophilic modification of a PTFE membrane with a polyglycerol-based UV-curable coating with fluoroalkyl hydrophobic segments for better adhesion on PTFE was patented.^26^ No description of the effect of the fluoroalkyl group on the adsorption of the coating was provided.

**Scheme 1:**
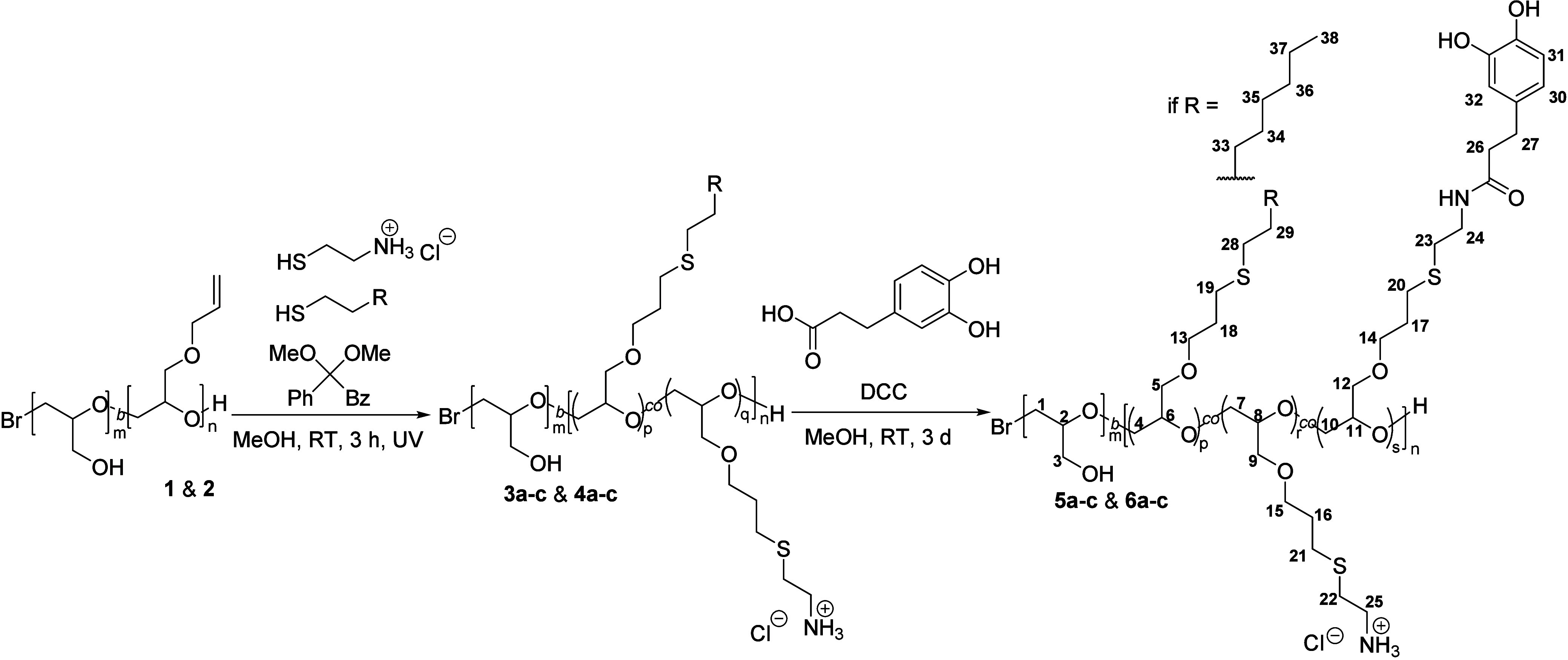
Synthesis of lPG Copolymers with Catechol and Hydrophobic or Fluorophilic Motifs in Their Side Chains Numbering of the atoms in the final product according to their assignment in the analysis of the NMR spectra.

This work elucidates the effect of fluorophilic and hydrophobic modification of catechol-derived polymers on their coating of low surface energy polymer surfaces, namely, PTFE and PS. Both materials find extensive use as biomaterials and in tissue culture where surface modification for lower protein adsorption is frequently encountered.^1,27−29^ Further, the coatings were compared to coatings on poly(methyl methacrylate) (PMMA), a more hydrophilic biomaterial.^28^ Two coating procedures were compared with each other: a classical catechol coating formation in Tris buffer at pH 8.5 and a two-step approach for a monolayer coating where the adsorption of the catechol-containing polymers was separated from the cross-linking step. A block copolymer of lPG with allyl glycidyl ether (AGE) was chosen as a platform to link the fluorophilic/hydrophobic anchoring and dopamine-derived adhesion moiety with a hydrophilic backbone to increase surface wettability (Scheme 1). Moreover, the tridecafluorooctyl-modified block copolymer was compared with a heptadecafluorodecyl-modified derivative, as fluorophilic interactions usually scale with increasing length of the fluoroalkyl group.

## Experimental Section

### Materials

Tetraoctyl ammonium bromide (98%), dry toluene (99.85%), calcium hydride (grains ≤2 mm, 93%), triisobutyl aluminum (1.1 M in toluene), toluene (analytical reagent grade), methanol (MeOH, ≥99.9%), acetone (≥99.8%), sodium chloride (≥99.5%), hydrochloric acid (HCl, 37 wt % in water), and diethyl ether (Et_2_O, ≥99%) were purchased from Acros Organics/AlfaAesar/Thermo Fisher Scientific (Schwerte, Germany). Molecular sieves (4 Å), tris(hydroxymethyl)aminomethane (Tris, 99.9%), HEPES (≥99.5%), and sodium sulfate (≥99%) were purchased from Carl Roth (Karlsruhe, Germany). Dichloromethane (DCM, ≈100%) was purchased from VWR (Darmstadt, Germany). 1-Octanethiol (98%) was purchased from Alfa Aesar (Haverhill, USA). Allyl glycidyl ether (AGE, >99%), 1*H*,1*H*,2*H*,2*H*-perfluorodecanethiol (>98%), and 2-aminoethanethiol hydrochloride (>95%) were bought from TCI (Eschborn, Germany). 3-(3,4-Dihydroxyphenyl)propionic acid (98%) was purchased from abcr (Karlsruhe, Germany). Dicyclohexyl carbodiimide (DCC, ≥99%), potassium persulfate (≥99.0%), fibrinogen from human plasma (product: F3879, 50–70% protein, ≥80% of protein is clottable), ethanol (≥99.8%), and dimethoxy phenyl acetophenone (DMPA, 99%) were purchased from Sigma-Aldrich/Merck Millipore (Taufkirchen, Germany). Magnesium sulfate (99%) was purchased from Grüssing (Filsum, Germany). Plates of polystyrene (PS; product number: 1122), poly(methyl methacrylate) (PMMA; product number: 1313), and polytetrafluoroethylene (PTFE; product number: 1703) were purchased with a thickness of 2 mm or 3 mm from s-Polytec (Kranenburg, Germany) and cut into 1 cm × 1 cm sized quadratic chips. Argon (Alphagaz, 99.999%) and nitrogen (Alphagaz, 99.999%) were purchased from Air Liquide (Düsseldorf, Germany). Deuterated methanol (99.8%) was purchased from Deutero (Kastellaun, Germany). 2-((1-Ethoxyethoxy)methyl)oxirane (EEGE) was synthesized according to a procedure of Fitton et al.^30^ with minor modifications (the reaction was cooled in an ice bath during the addition of *para*-toluenesulfonic acid monohydrate; extraction with sodium bicarbonate solution was performed three times instead of once). AGE and EEGE were dried with calcium hydride at room temperature overnight and distilled in high vacuum onto a dried molecular sieve (4 Å) prior to use. The synthesis of 1*H*,1*H*,2*H*,2*H*-perfluorooctanethiol was performed according to the procedure of S. Y. Dieng et al.^31^ The only two modifications were that the second reaction step (hydrolysis) was performed for 4 h at 100 °C and that the final product was extracted with DCM. Spectra/Por membranes (regenerated cellulose, molecular weight cutoff: 1 kDa) from Repligen (Waltham, USA) were used for dialysis.

### Nuclear Magnetic Resonance (NMR)

1D-NMR spectra were recorded on an ECX400 instrument (399.75 MHz ^1^H frequency; 376.14 MHz ^19^F frequency; 9.39 T) from JEOL (Freising, Germany). 2D-NMR spectra were recorded on an ECZ600 (600.17 MHz ^1^H frequency; 564.73 MHz ^19^F frequency; 150.91 MHz ^13^C frequency; 14.09 T) from JEOL. MestReNova version 14.1.1-24571 from Mestrelab Research (Santiago de Compostela, Spain) was used to display and process the spectra. The raw data files were opened using the default settings. 1D-NMR spectra were exponentially apodized (^1^H: 0.5 Hz, ^19^F: 5.0 Hz), referenced to methanol (3.31 ppm; only ^1^H spectra), if necessary, manually phase corrected, and baseline corrected using the Whittaker smoother function. 2D-NMR spectra were apodized along f1 and f2 using the sine square (0.00°) and sine square II function (0.0% and 50.0%) and baseline corrected in all dimensions using the Whittaker smoother function. The degrees of functionalization were determined by calculating the ratio of the integrals of characteristic signals of the different repeating units. The signal between 6.7 and 6.6 ppm was taken as a reference signal for catechol groups. The signal between 3.2 and 3.1 ppm was taken as a reference signal for amine groups. The signal between 2.5 and 2.4 ppm was taken as a reference signal for fluoroalkyl groups (after subtracting the integral of the catechol reference signal). The signal between 1.65 and 1.55 ppm was taken as a reference signal for alkyl groups.

### Water Contact Angle (WCA)

The (static) WCA was determined using an OCA 20 instrument from Dataphysics Instruments GmbH (Filderstadt, Germany) equipped with a charge-coupled device (CCD) camera. Four drops of Milli-Q water (2 μL) were placed on each sample surface. The integrated software was used to measure the contact angle of the water droplet by a two-sided tangential approximation. The mean of both tangent angles was used as the contact angle of the respective drop.

### Scanning Electron Microscopy (SEM)

Scanning electron microscopy was performed on an SU8030 instrument from Hitachi (Düsseldorf, Germany). The samples were adhered to a copper band and sputtered with gold (5 nm) using a CCU-010 compact coating unit from Safematic (Zizers, Switzerland). A current of 10 μA and an acceleration voltage of 15 kV were used for the electron beam. The beam, apparatus, and X and Y were aligned to minimize the movement of the image. Images of 1280 × 960 pixels size were recorded.

### Dynamic Light Scattering (DLS)

DLS was measured using a Zetasizer Nano ZS from Malvern Panalytical (Malvern, UK) with a red laser in 173° back scattering mode in 10 mm × 10 mm UV quartz cuvettes from Carl Roth (article number: X854.1) at 25 °C after 30 s of equilibration. Polystyrene latex with a refractive index (RI) of 1.590 and adsorption of 0.010 was chosen as the material, and the dispersant properties were calculated with the Zetasizer software (version 7.11) from Malvern Panalytical. The dispersant depending on the sample was either methanol (η = 0.5476 cP, RI = 1.326) or water/methanol 1/1 (η = 1.7047 cP, RI = 1.340). Each sample was measured three times with 11 scans (*t* = 10 s). Solutions of 9/1-Oct-Cat **6a** or 1/1-FOct-Cat **5b** in either pure methanol or a *v*/*v* 1/1 mixture of methanol and Milli-Q water (*c* = 0.01 mg mL^–1^ to 10 mg mL^–1^) were prepared for studying their aggregation behavior similar to the procedure of Skhiri et al.^32^ The solutions were filtered through a 0.2 μm regenerated cellulose filter prior to the measurement. The logarithmic of the mean-derived count rate was plotted against the logarithmic concentration of the polymer solution.

### Quartz-Crystal Microbalance with Dissipation (QCM-D)

Quartz-crystal microbalance with dissipation was performed on a QSense from Biolin Scientific (Gothenburg, Sweden) using an ISM596D peristaltic pump from Ismatec (Wertheim, Germany). The preparation of the titanium sensors is described in the ellipsometry section (Supporting Information). All measurements were performed following the procedure of Yu et al.^33^ in general with a set flow rate of 0.07 mL min^–1^, which equaled a real flow rate of approximately 0.1 mL min^–1^. HEPES buffer (0.1 mmol L^–1^ 2-[4-(2-hydroxyethyl)piperazinyl]ethanesulfonic acid, 0.15 mmol L^–1^ sodium chloride, adjusted to pH 7.4) was pumped over the sensor until baseline stabilization. After recording a baseline for 150–180 s, the feed solution was changed from HEPES buffer to a fibrinogen solution in HEPES buffer (1 mg mL^–1^) for 30 min (the solution takes some time to reach the sensor). Afterward, the sensors were washed with HEPES buffer for 15 min.

### Syntheses

The syntheses were performed following modified procedures from Yu et al.^17^ The synthesis procedure of the two base polymers **1** and **2** originates from Gervais et al.^34^

### Polymerization (**1** and **2**)

Educt and product weights can be found in Table S1. Tetraoctyl ammonium bromide (1.0 equiv) was dried by repeatedly melting it in a Schlenk flask under vacuum. Dry toluene (9/1-copolymer: 96 mL or 1/1-copolymer: 144 mL) was added. The mixture was sonicated to solubilize the salt. Dry EEGE (1/1-copolymer: 9.0 equiv or 9/1-copolymer: 81 equiv) and triisobutyl aluminum solution (3.0 equiv) were added, and the mixture was stirred for 4 h in an ice bath. AGE (9.0 equiv) and triisobutyl aluminum solution (1.0 equiv) were added, and the mixture was stirred at room temperature overnight. The reaction was terminated by the addition of water (2 mL) at 0 °C and dried with magnesium sulfate (4.00 g). The mixture was centrifuged to remove the solids and concentrated in vacuum. Upon removal of the toluene, the raw product was solubilized in diethyl ether (40 mL) and stored in the freezer upon precipitation of a solid. The solid was again removed in the centrifuge, and the raw product concentrated in vacuum. Dialysis against acetone for 5 d and distillation of the solvent gave the intermediary polymer as a colorless (9/1-copolymer) or orange-brown (1/1-copolymer) slimy solid. The polymers were dissolved in ethanol (30 mL), and hydrochloric acid (37 wt %; 0.25 mL) was added. The reaction mixture was stirred for 1 d at room temperature before it was concentrated. The residue was dissolved in ethanol (30 mL) and hydrochloric acid (37 wt %; 0.25 mL) again, stirred for 2 d at room temperature, and concentrated. The repetition of the hydrolysis ensures full conversion in our experience. Dialysis against methanol for 5 days gave polymer **1** or **2** as a colorless gel (9/1-copolymer) or brown oil (1/1-copolymer). 1/1-copolymer **1**: **GPC**: *M*_*n*_ = 7.09 kDa, *M*_*w*_ = 7.57 kDa; *Đ* = 1.07. ^**1**^**H NMR** (600 MHz, CD_3_OD): δ = 5.92 (ddt, *J* = 17.2 Hz, 10.7 Hz, 5.3 Hz, alkene, 1H), 5.29 (d, *J* = 17.1 Hz, alkene, 1H), 5.16 (d, *J* = 10.6 Hz, alkene, 1H), 4.01 (d, *J* = 5.4 Hz, allyl position, 2H), 3.74–3.47 (m, backbone, 5.4H) ppm. 9/1-copolymer **2**: **GPC**: *M*_*n*_ = 10.1 kDa, *M*_*w*_ = 11.6 kDa; *Đ* = 1.14. ^**1**^**H NMR** (600 MHz, CD_3_OD): δ = 5.92 (ddt, *J* = 17.2 Hz, 10.8 Hz, 5.4 Hz, alkene, 1H), 5.29 (d, *J* = 17.2 Hz, alkene, 1H), 5.17 (d, *J* = 10.5 Hz, alkene, 1H), 4.04–4.00 (m, allyl position, 2H), 3.77–3.49 (m, backbone, 44H) ppm.

### Thiol–Ene Coupled Intermediates (**3a**–**c** and **4a**–**c**)

Educt and product weights can be found in Table S2. Polymer **1** or **2** (1 equiv of allyl groups) was dissolved in methanol (25 mL). 2-Aminoethanethiol hydrochloride (2.67 equiv) and the respective thiol (1.33 equiv) were added to the solution, and the reaction mixture was stirred for 3 h at room temperature under UV irradiation from a PR160L-370 nm Gen2 lamp (λ = 370 nm, *I* = 399 mW cm^–2^) from Kessil (Richmond, USA). After 0, 1, and 2 h reaction time, a solution of DMPA in methanol (concentration: 100 g L^–1^; 0.02 equiv each time) was added. The stepwise addition of the initiator reinitiates the reaction twice and decreases the concentration of radicals, which both contribute to a complete conversion. The mixture was transferred into dialysis tubing and dialyzed against methanol for 5–7 d. The fluorinated polymer solutions were filtered once to remove some precipitate. Distillation of the solvent gave the intermediary polymers as colorless to yellow, sticky solids. Noteworthy, the fluorinated polymers especially with a heptadecafluorodecyl group have a very high tendency to foam under reduced pressure. 1/1-Oct **3a**: ^**1**^**H NMR** (400 MHz, CD_3_OD): δ = 3.78–3.42 (m, H-1–H-9, H-13, H-15), 3.21–3.11 (m, H-28), 3.00–2.75 (m, H-25), 2.75–2.65 (m, H-19, H-21), 2.65–2.57 (m, H-19), 2.57–2.41 (m, H-22), 1.95–1.72 (m, H-16, H-18), 1.65–1.53 (m, H-29), 1.53–1.19 (m, H-33–H-37), 0.98–0.79 (m, H-38) ppm. 1/1-FOct **3b**: ^**1**^**H NMR** (600 MHz, CD_3_OD): δ = 3.79–3.44 (m, H-1–H-9, H-13, H-15), 3.22–3.13 (m, H-28), 2.92–2.81 (m, H-25), 2.81–2.75 (m, H-22), 2.75–2.65 (m, H-19, H-21), 2.55–2.41 (m, H-29), 1.94–1.83 (m, H-16, H-18) ppm. ^**19**^**F NMR** (565 MHz, CD_3_OD): δ = −82.3 (3F), −115.1 (2F), −122.8 (2F), −123.8 (2F), −124.1 (2F), −127.2 (2F) ppm. 1/1-FDec **3c**: ^**1**^**H NMR** (600 MHz, CD_3_OD): δ = 3.80–3.45 (m, H-1–H-9, H-13, H-15), 3.21–3.13 (m, H-28), 2.91–2.82 (m, H-25), 2.82–2.74 (m, H-22), 2.74–2.64 (m, H-19, H-21), 2.55–2.36 (m, H-29), 1.95–1.74 (m, H-16, H-18) ppm. ^**19**^**F NMR** (565 MHz, CD_3_OD): δ = −82.3 (3F), −115.1 (2F), −122.8 (6F), −124.1 (4F), −127.2 (2F) ppm. 9/1-Oct **4a**: ^**1**^**H NMR** (400 MHz, CD_3_OD): δ = 3.80–3.43 (m, H-1–H-9, H-13, H-15), 3.21–3.11 (m, H-28), 2.90–2.78 (m, H-25), 2.73–2.65 (m, H-19, H-21), 2.65–2.57 (m, H-19), 2.57–2.49 (m, H-22), 1.94–1.80 (m, H-16, H-18), 1.64–1.54 (m, H-29), 1.46–1.27 (m, H-33–H-37), 0.97–0.84 (m, H-38) ppm. 9/1-FOct **4b**: ^**1**^**H NMR** (600 MHz, CD_3_OD): δ = 3.79–3.46 (m, H-1–H-9, H-13, H-15), 3.21–3.13 (m, H-28), 2.89–2.81 (m, H-25), 2.81–2.74 (m, H-22), 2.72–2.66 (m, H-19, H-21), 2.54–2.41 (m, H-29), 1.93–1.83 (m, H-16, H-18) ppm. ^**19**^**F NMR** (565 MHz, CD_3_OD): δ = −82.3 (3F), −115.1 (2F), −122.8 (2F), −123.8 (2F), −124.1 (2F), −127.2 (2F) ppm. 9/1-FDec **4c**: ^**1**^**H NMR** (600 MHz, CD_3_OD): δ = 3.81–3.49 (m, H-1–H-9, H-13, H-15), 3.20–3.13 (m, H-28), 2.89–2.81 (m, H-25), 2.81–2.73 (m, H-22), 2.73–2.67 (m, H-19, H-21), 2.53–2.36 (m, H-29), 1.94–1.84 (m, H-16, H-18) ppm. ^**19**^**F NMR** (565 MHz, CD_3_OD): δ = −82.8 (3F), −115.2 (2F), −122.9 (6F), −124.1 (4F), −127.6 (2F) ppm.

### Amide Condensation to Final Coating Polymers (**5a**–**c** and **6a**–**c**)

Educt and product weights can be found in Table S3. The respective polymer **3a**–**c** or **4a**–**c** (1 equiv of amine groups), DCC (3.0 equiv), and 3-(3,4-dihydroxyphenyl)propionic acid (3.0 equiv) were dissolved in methanol (25 mL). The reaction was stirred under an argon atmosphere in the dark at room temperature for 3 d. The mixture was transferred into dialysis tubing and dialyzed against methanol for 4–7 d in the dark. The precipitate was removed by either filtration or centrifugation. Distillation of the solvent gave the final coating polymers as yellow to light brown, sticky solids. Noteworthy, the fluorinated polymers especially with a heptadecafluorodecyl group have a very high tendency to foam under reduced pressure. Due to the detection of impurities in the NMR spectra of polymers **5b** and **5c**, these two polymers were dissolved in methanol (10 mL) again, centrifuged, and dialyzed in methanol under the exclusion of light for a further 6 d. 1/1-Oct-Cat **5a**: ^**1**^**H NMR** (400 MHz, CD_3_OD): δ = 6.71–6.63 (m, H-31, H-32), 6.55–6.50 (m, H-30), 3.79–3.41 (m, H-1–H-15), 3.18–3.12 (m, H-28), 2.97–2.71 (m, H-25, H-26), 2.71–2.47 (m, H-19–H-23), 2.47–2.37 (m, H-27), 1.92–1.71 (m, H-16–H-18), 1.64–1.52 (m, H-29), 1.52–1.21 (m, H-33–H-37), 0.97–0.84 (m, H-38) ppm. H-24 not visible in the 1D-^1^H NMR spectrum since it overlays with solvent signal. 1/1-FOct-Cat **5b**: ^**1**^**H NMR** (400 MHz, CD_3_OD): δ = 6.74–6.60 (m, H-31, H-32), 6.57–6.46 (m, H-30), 3.83–3.39 (m, H-1–H-15), 3.19–3.10 (m, H-28), 2.87–2.72 (m, H-22, H-25, H-26), 2.72–2.51 (m, H-19–H-21, H-23), 2.51–2.34 (m, H-27, H-29), 1.93–1.75 (m, H-16–H-18) ppm. H-24 not visible in the 1D-^1^H NMR spectrum since it overlays with solvent signal. ^**19**^**F NMR** (376 MHz, CD_3_OD): δ = −82.3 (3F), −115.1 (2F), −122.8 (2F), −123.8 (2F), −124.1 (2F), −127.2 (2F) ppm. 1/1-FDec-Cat **5c**: ^**1**^**H NMR** (400 MHz, CD_3_OD): δ = 6.75–6.62 (m, H-31, H-32), 6.56–6.46 (m, H-30), 3.83–3.41 (m, H-1–H-15), 3.23–3.09 (m, H-28), 2.93–2.72 (m, H-22, H-25, H-26), 2.72–2.51 (m, H-19–H-21, H-23), 2.51–2.32 (m, H-27, H-29), 1.94–1.74 (m, H-16–H-18) ppm. H-24 not visible in the 1D-^1^H NMR spectrum since it overlays with solvent signal. ^**19**^**F NMR** (376 MHz, CD_3_OD): δ = −82.2 (3F), −115.1 (2F), −122.8 (6F), −124.0 (4F), −127.2 (2F) ppm. 9/1-Oct-Cat **6a**: ^**1**^**H NMR** (400 MHz, CD_3_OD): δ = 6.71–6.63 (m, H-31, H-32), 6.56–6.51 (m, H-30), 3.79–3.46 (m, H-1–H-15), 3.19–3.10 (m, H-28), 2.97–2.73 (m, H-25, H-26), 2.71–2.48 (m, H-19–H-23), 2.48–2.38 (m, H-27), 1.94–1.72 (m, H-16–H-18), 1.64–1.53 (m, H-29), 1.46–1.22 (m, H-33–H-37), 0.98–0.83 (m, H-38) ppm. H-24 not visible in the 1D-^1^H NMR spectrum since it overlays with solvent signal. 9/1-FOct-Cat **6b**: ^**1**^**H NMR** (400 MHz, CD_3_OD): δ = 6.73–6.61 (m, H-31, H-32), 6.56–6.49 (m, H-30), 3.82–3.40 (m, H-1–H-15), 3.20–3.10 (m, H-28), 2.93–2.72 (m, H-22, H-25, H-26), 2.72–2.53 (m, H-19–H-21, H-23), 2.53–2.36 (m, H-27, H-29), 1.95–1.76 (m, H-16–H-18) ppm. H-24 not visible in the 1D-^1^H NMR spectrum since it overlays with solvent signal. ^**19**^**F NMR** (376 MHz, CD_3_OD): δ = −82.3 (3F), −115.1 (2F), −122.8 (2F), −123.8 (2F), −124.1 (2F), −127.2 (2F) ppm. 9/1-FDec-Cat **6c**: ^**1**^**H NMR** (400 MHz, CD_3_OD): δ = 6.75–6.60 (m, H-31, H-32) 6.55–6.47 (m, H-30), 3.83–3.41 (m, H-1–H-15), 3.18–3.10 (m, H-28), 2.85–2.50 (m, H-19–H-23, H-25, H-26), 2.50–2.30 (m, H-27, H-29), 1.92–1.74 (m, H-16–H-18) ppm. H-24 not visible in the 1D-^1^H NMR spectrum since it overlays with solvent signal. ^**19**^**F NMR** (376 MHz, CD_3_OD): δ = −82.2 (3F), −115.1 (2F), −122.8 (6F), −124.0 (4F), −127.2 (2F) ppm.

### Coating Procedure

The cleaned and degreased polymeric surfaces (with acetone and methanol) were placed in a PS Petri dish (diameter: 35 mm), and 2.5 mL of a 2 mg mL^–1^ solution of the polymer in methanol was filled into the Petri dish. A 2.5 mL amount of an aqueous tris(hydroxymethyl)aminomethane solution (1 mol L^–1^) that was adjusted to pH 8.5 by adding hydrochloric acid (0.33 mol L^–1^) was added after 15 min. The surfaces were washed with methanol and dried in a stream of nitrogen for 24 h.

### Monolayer Coating Procedure

The procedure was adapted from Yu et al.^17^ The cleaned and degreased polymeric surfaces (with acetone and methanol) were placed in a PS Petri dish (diameter: 35 mm), and 5 mL of a 0.02 mg mL^–1^ solution of the polymer in methanol was filled into the Petri dish. The samples were rinsed with methanol after 2 h and dried in a stream of nitrogen. The surfaces were then immersed in 5 mL of an aqueous solution of tris(hydroxymethyl)aminomethane (1 mol L^–1^) that was adjusted to pH 8.5 by adding hydrochloric acid (0.33 mol L^–1^) and potassium persulfate (10 mg g^–1^). The surfaces were washed with water and methanol and dried in a stream of nitrogen after 1 h.

## Results and Discussion

An lPG/AGE block copolymer with an lPG to AGE molar ratio of 9, *M*_*n*_ of 10.1 kDa, *M*_*w*_ of 11.6 kDa, and dispersity of 1.14 (Figures S1 and S2), and another polymer with an lPG to AGE molar ratio of 1, *M*_*n*_ of 7.1 kDa, *M*_*w*_ of 7.6 kDa, and dispersity of 1.07 (Figures S3 and S4), respectively, were selected as starting materials for the functionalization with three different hydrophobic groups each (Scheme 1 and Table 1). Each type of repeating unit of the targeted copolymeric structure fulfills a designated role. Polyglycerol provides hydrophilicity as well as biocompatibility,^5,6^ catechol is the oxidizable cross-linking unit, amine groups facilitate the cross-linking,^17^ and the octyl, tridecafluorooctyl, or heptadecafluorodecyl unit modifies the polymer hydrophobically or fluorophilically. Use of block copolymers over random or alternating copolymers leads to the formation of a more hydrophobic anchoring block and a more hydrophilic lPG block to increase the surface wettability. Albeit a desired structural design for the coating of hydrophobic surfaces, this induces pronounced amphiphilic behavior to the polymers after the first functionalization step. Especially the fluoroalkylated polymers **3**–**6b**,**c** have a strong to extreme tendency to stabilize gas bubbles inside of solutions, which needs to be considered especially during distillations. The synthesis started with a UV light-initiated thiol-alkene click reaction succeeded by an amide coupling following the general synthetic concept of Yu et al.^17^ However, two thiols, cysteamine and a (fluoro)alkyl thiol, were added simultaneously to the AGE block in a 2 to 1 molar ratio, resulting in the formation of a bifunctional AGE block. The ratio of cysteamine groups to (fluoro)alkyl groups in the intermediate roughly corresponds to the 2 to 1 ratio (Table S4), while no allyl groups can be detected afterward in the NMR spectra (Figures S5–S14).

**Table 1:** Overview of Polymers Used for the Coating of PTFE, PS, and PMMA

**Polymer**	**Code**	***m*/*n***	**R**
1/1-Oct(-Cat)	**3a** and **5a**	1	C_6_H_13_
1/1-FOct(-Cat)	**3b** and **5b**	1	C_6_F_13_
1/1-FDec(-Cat)	**3c** and **5c**	1	C_8_F_17_
9/1-Oct(-Cat)	**4a** and **6a**	9	C_6_H_13_
9/1-FOct(-Cat)	**4b** and **6b**	9	C_6_F_13_
9/1-FDec(-Cat)	**4c** and **6c**	9	C_8_F_17_

**Figure 1:**
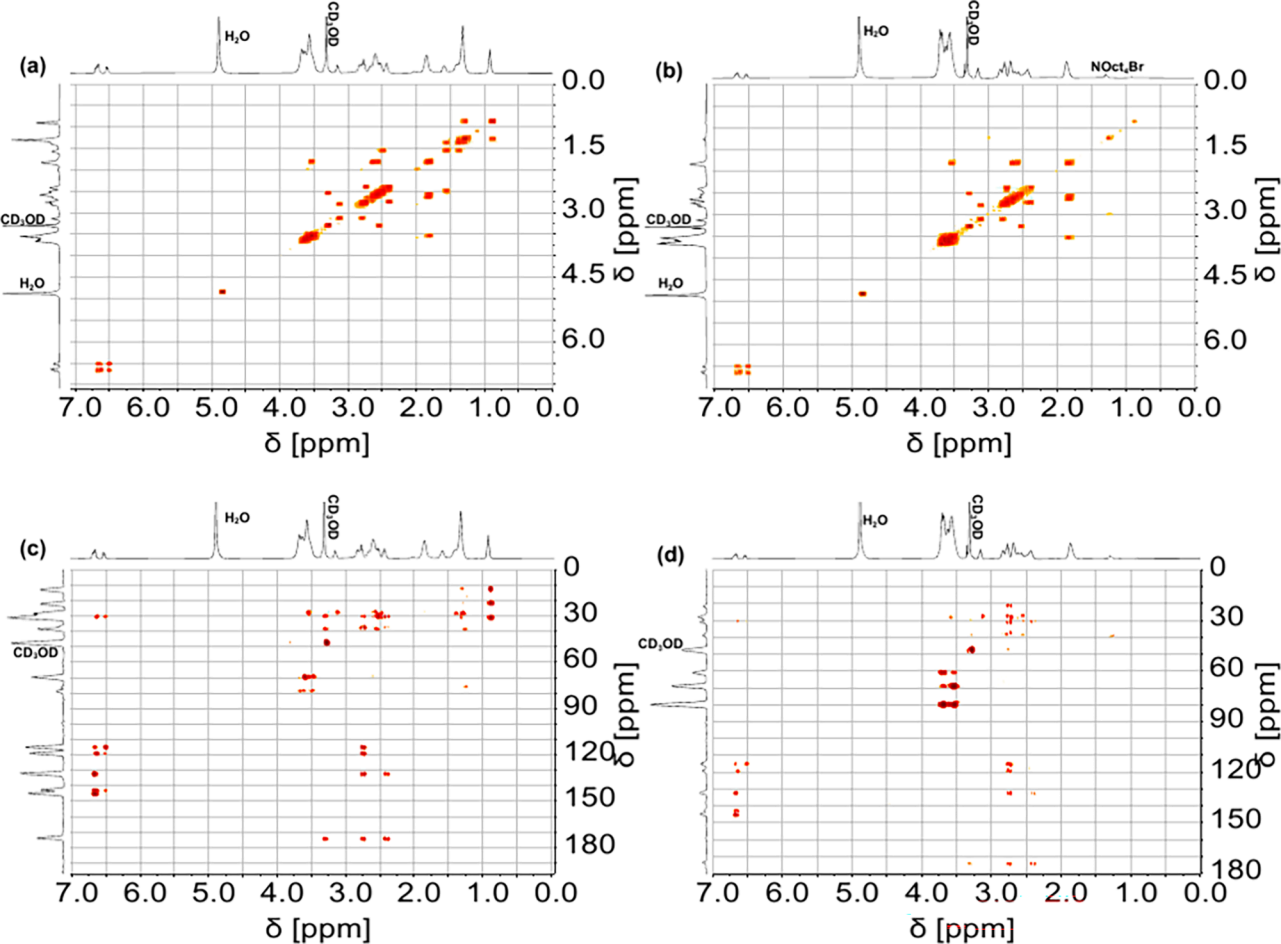
^1^H,^1^H-COSY NMR spectra of (a) 1/1-Oct-Cat **5a** and (b) 9/1-FOct-Cat **6b**. ^1^H,^13^C-HMBC NMR spectra of (c) 1/1-Oct-Cat **5a** and (d) 9/1-FOct-Cat **6b**. All spectra were recorded at 600 MHz (^1^H frequency) in CD_3_OD.

Amide coupling between the amine groups of the cysteamine and carboxylic groups of 3-(3,4-dihydroxyphenyl)propanoic acid was facilitated by DCC under neutral conditions. Conversion of amine groups to amides is purposely incomplete under these conditions because unreacted amine groups at the polymer can facilitate the cross-linking process in basic conditions.^17^ The final coating polymers are transparent to yellow sticky or stiff solids that are almost insoluble in water, DMF, and THF but show good solubility in methanol. This rendered attempts to characterize their increase in molecular weight by gel permeation chromatography unsuccessful. Instead, ^1^H,^1^H–COSY and ^1^H,^13^C-HMBC NMR spectroscopy (Figure 1, 1D NMR: Figures S15–S24) was used to confirm the structure of the polymers and estimate their comparable degree of functionalization (Table S5).

PS, PTFE, and PMMA slides were coated by immersing them into a methanolic solution (2 mg mL^–1^) of the respective coating polymer. Addition of tris(hydroxymethyl)aminomethane (Tris) buffer (pH = 8.5) initiated the cross-linking of dopamine. Upon addition of the aqueous buffer, the solution became turbid immediately. Coating of the polymers using the more hydrophilic polymers **6a**–**c** did not lead to visual changes. Contrastingly, coating with the more hydrophobic polymers **5a**–**c** led to the formation of a dopamine-typical brownish^13^ coating layer (Figure S25), which is consistent with their higher content of catechol groups. For example, the coatings of polymer 1/1-FOct-Cat **5b** on PS/titanium-coated quartz crystal microbalance (QCM) sensors were so nontransparent or thick that their thicknesses could not be determined by spectroscopic ellipsometry. For comparison, polymer 9/1-Oct-Cat **6a** formed transparent coatings with a thickness of only (8 ± 3) nm under the same conditions.

**Figure 2:**
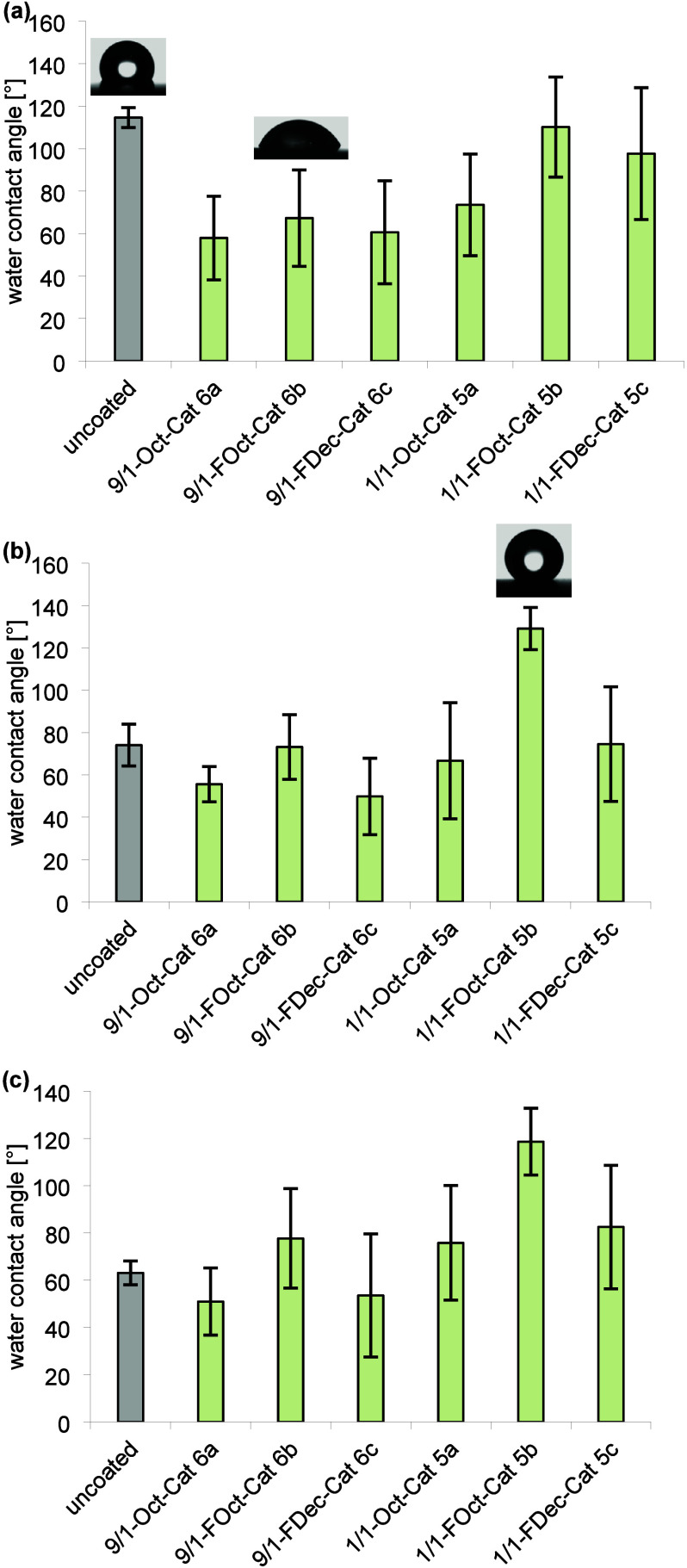
WCAs of coated (a) PTFE, (b) PS, and (c) PMMA. Each bar represents the mean and standard deviation of 24 measurements (four spots per surface, three surfaces in separate coating solutions, replicated on a second day).

**Figure 3:**
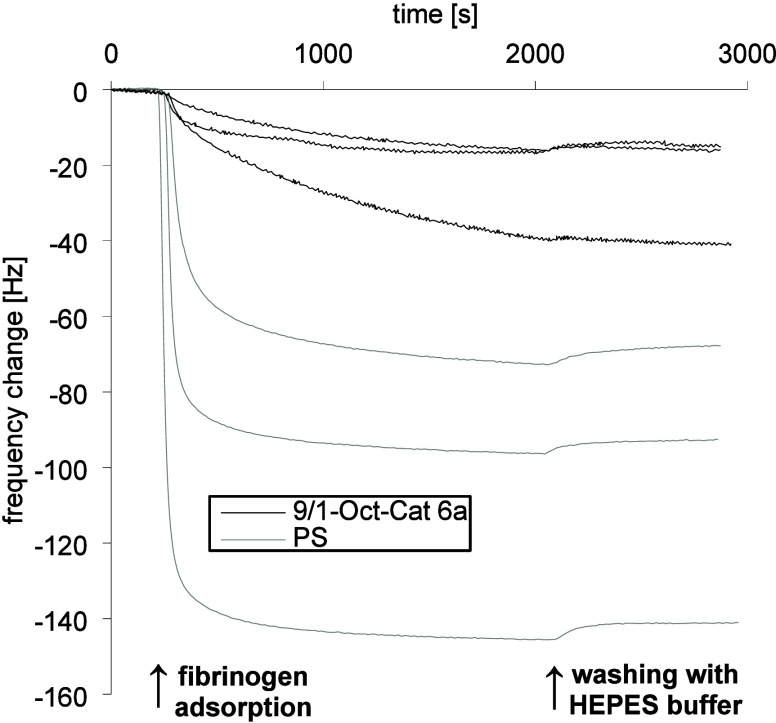
Frequency change of the third overtones during the adsorption of fibrinogen onto PS/titanium-coated quartz sensors (*n* = 3) without further coating and with an additional coating with 9/1-Oct-Cat **6a**.

Interestingly, coatings of the more hydrophilic polymers **6a**–**c** on PS, PTFE, and PMMA possess similar WCAs on average around 60° independent of the hydrophobic or fluorophilic group of the coating polymer (Figure 2), apart from the WCAs of 9/1-FOct-Cat **6b** on PS (73 ± 15)° and PMMA (78 ± 21)°, which were significantly (α = 0.05) higher than WCAs of coatings of 9/1-Oct-Cat **6a** and 9/1-FDec-Cat **6c** on PS and PMMA, respectively (as confirmed by a two-sample Wilcoxon rank sum test or a one-way χ^2^ approximated Kruskal–Wallis test using JMP 16.0.0). This indicates that the hydrophobic or fluorophilic units of polymers **6a**–**c** are likely oriented predominantly toward the substrate’s surface. The reduction of the WCAs of PS and PTFE substrates upon coating with polymers **6a**–**c** and **5a** (PTFE) and **6a**, **6c**, and **5a** (PS) inspired us to study the adsorption of fibrinogen from human plasma on PS/titanium-coated quartz sensors before and after an additional coating with 9/1-Oct-Cat **6a** using QCM-D. Sensors that were coated with 9/1-Oct-Cat **6a** (Figure 3, dissipation plot: Figure S26) showed a smaller change in frequency of the third overtone upon contact with fibrinogen than the native PS/titanium-coated quartz sensors. This observation suggests a decreased fibrinogen adsorption and, thus, an increased hydrophilicity and anti-biofouling performance upon coating. What strikes the eye are the high WCAs (up to (129 ± 10)° on PS) of coatings formed by 1/1-FOct-Cat **5b**. This observation suggests that the fluoroalkyl groups of **5b** orient at least partially away from the substrate surface to form a typical fluorous hydrophobic coating^1,27,35^ on all surfaces independently of the fluorophilicity of the surface. Coating with polymer 1/1-FDec **5c** leads to notably lower WCAs (only (75 ± 27)° on PS) than **5b** despite the longer fluoroalkyl group attached to the coating. Further, PS and to some extent PMMA showed partial detachment of the coating in the washing step, while coatings on PTFE did not change visually during the washing (Figures S25, S27, and S28). The WCAs’ overall stronger dependence on the coating’s hydrophobic/fluorophilic modification in the case of the more hydrophobic polymers **5a**–**c** is as expected since these coating polymers contain a relatively higher fraction of hydrophobic/fluorophilic tags than polymers **6a**–**c**.

**Figure 4:**
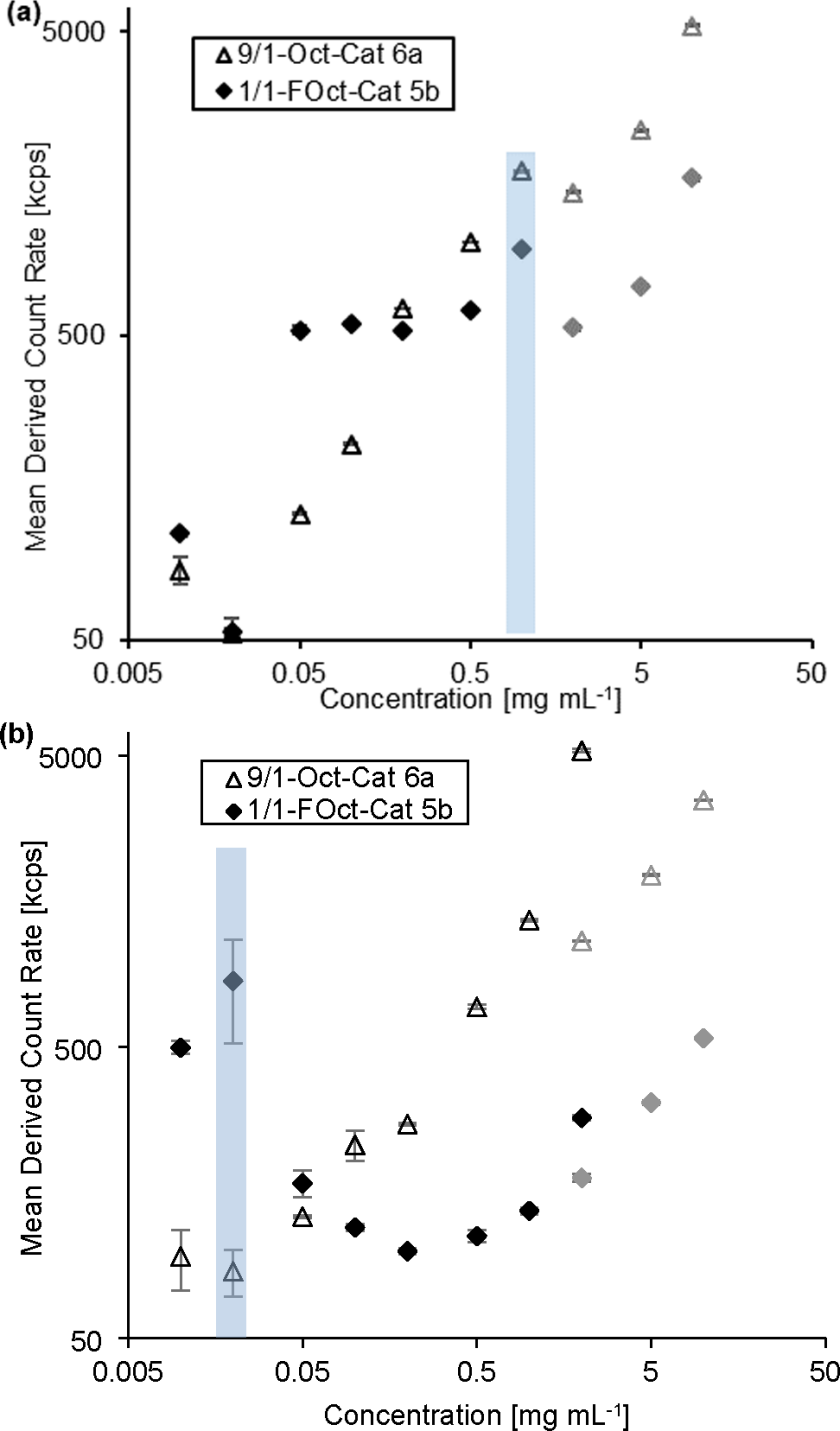
Mean derived count rate in kilocounts per second (kcps) during a DLS measurement in dependence on the concentration of 9/1-Oct-Cat **6a** and 1/1-FOct-Cat **5b** in (a) 50 vol % methanol in water and (b) in methanol. Standard deviation of the three (six in the case of the two lowest concentrations of **5b** in MeOH) measurements is represented with error bars. Gray points at 2–10 mg mL^–1^ above the relevant coating concentrations were created from a different stock solution and show lower than expected derived count rates but display an increasing trend of the count rate. Mean derived count rate of MeOH (without polymer): (230 ± 40) kcps and (540 ± 50) kcps of 50 vol % methanol in water. The blue bar marks the concentrations relevant for the coating processes.

**Figure 5:**
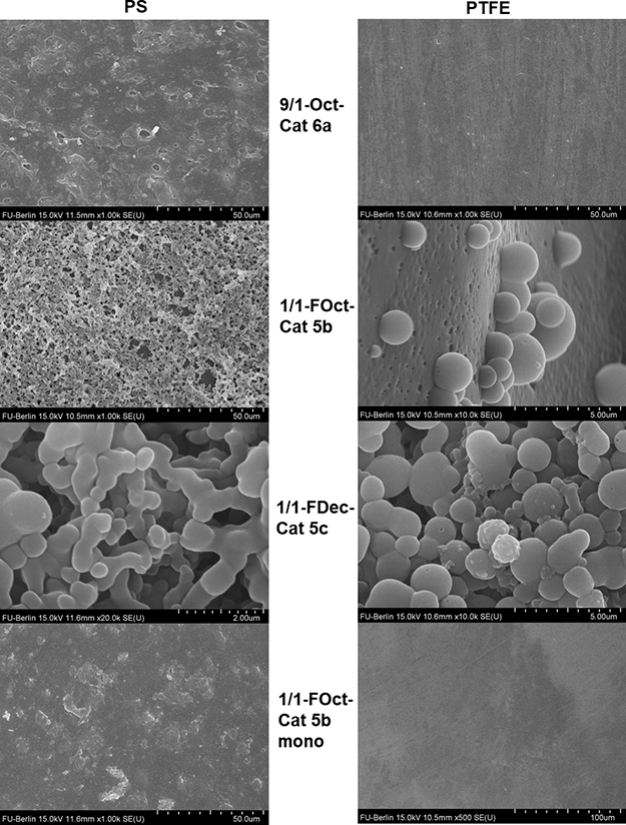
Overview of SEM images of coatings on PTFE and PS at different magnifications. Further SEM images can be found in the Supporting Information (Figures S33–S42).

We hypothesized that the relatively small differences between the WCA values of most surface coatings might arise from an aggregation of the polymers during the coating process. Aggregates would have their hydrophilic segments positioned toward polar solvents, thus hindering the hydrophobic/fluorophilic moiety to interact with the solvent and the substrates. Aggregation of dopamine-based coatings is a well-documented phenomena and can drastically alter the resulting coatings’ properties;^36−41^ for example, topologically structured surfaces that are formed as a result of aggregation during the cross-linking process can promote cell adhesion and mechanosensing.^42^ However, structurally similar lPG-catechol block copolymers with similar catechol content exhibited a stronger tendency to form thin, smooth coatings rather than aggregate-driven, thick and rough coatings.^8^ The aggregation behavior of the most hydrophilic coating polymer 9/1-Oct-Cat **6a** and the most hydrophobic 1/1-FOct-Cat **5b** (at least according to the wettability of the resulting coatings in Figure 2) was studied using DLS to test this hypothesis (Figure 4a, hydrodynamic radii: Figures S29–S32). It was assumed that the other polymers would probably possess an aggregation behavior among these two polymers. Methanol (50 vol %) in water was used as solvent since methanolic Tris buffer triggers immediate cross-linking. An increase of the mean derived count rate of a solution is a good indicator for the presence of aggregates.^32^ Polymer 9/1-Oct-Cat **6a** and 1/1-FOct-Cat **5b** start aggregating latest around 0.5 mg mL^–1^, which demonstrates the existence of aggregates in the coating process (concentration of the polymers: 1 mg mL^–1^). Formation of spherical to spheroidal particles of 1/1-FOct-Cat **5b** on PS and PTFE with a diameter of 1–2 μm upon cross-linking was confirmed by SEM (Figures 5, S33, and S34). The images show a base layer of rather smooth coating on PTFE with spherical particles being adsorbed on the topmost layer. Similar images were obtained for the coating of 1/1-FDec-Cat **5c** on PS and PTFE (Figures 5, S35, and S36). Coatings of 9/1-Oct-Cat **6a** (Figures 5, S37, and S38) are, however, barely distinguishable from the uncoated substrates (Figures S39 and S40), which is consistent with their small thickness detected by ellipsometry. Most investigated polymers except for 1/1-FOct-Cat **5b** form generally similar coatings on PTFE and PS by means of SEM.

**Figure 6:**
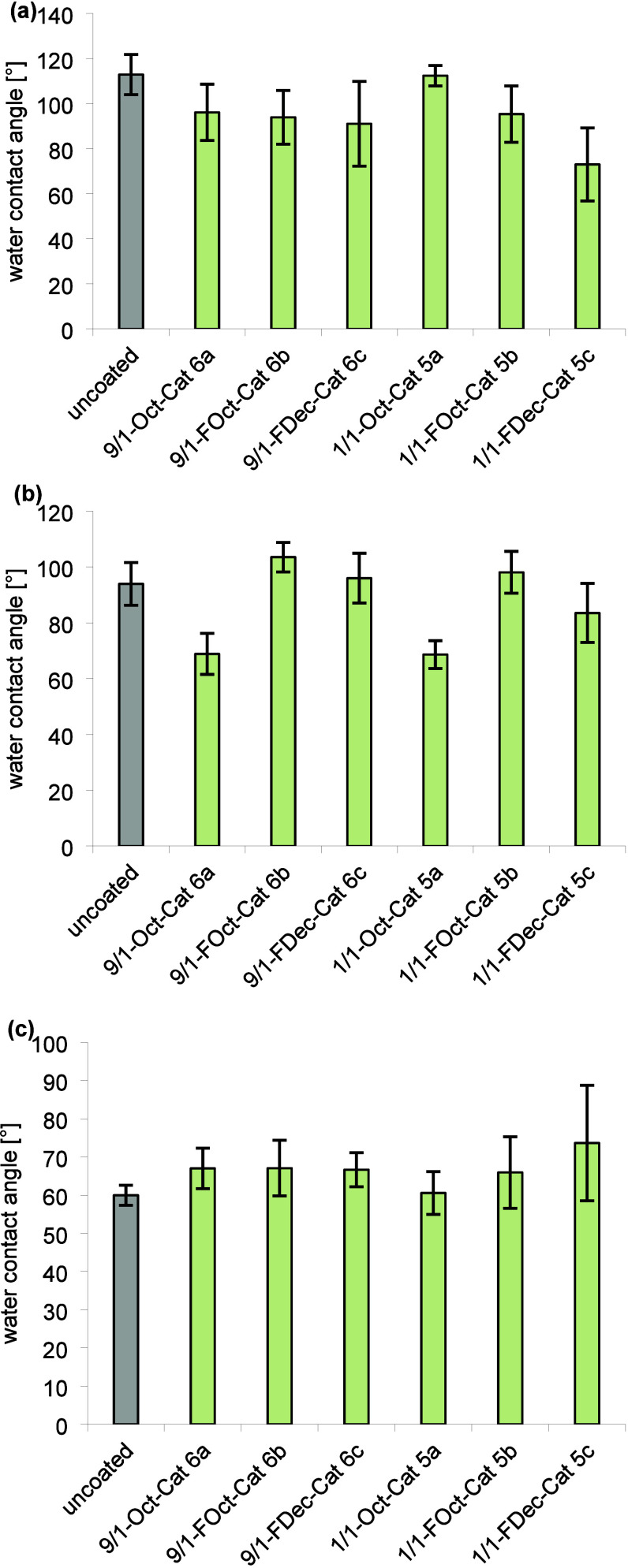
WCAs of a monolayer on (a) PTFE, (b) PS, and (c) PMMA. Each bar represents the mean and standard deviation of 24 measurements (four spots per surface, three surfaces in separate coating solutions, replicated on a second day).

A coating process that was used in the past to form lPG monolayers by catechol cross-linking chemistry^17^ was used in a second series of coatings to exclude aggregation effects during the coating process. The substrates were immersed in a methanolic solution of the coating polymer to initiate the adsorption of only a thin film onto the surface. The substrates were washed afterward and immersed in a basic Tris buffer that contained potassium persulfate as oxidizer. A second series of DLS measurements in pure methanol was performed to evaluate the aggregation of 1/1-FOct-Cat **5b** and 9/1-Oct-Cat **6a** in the first adsorption step (Figure 4b). It was found that a concentration as low as 0.02 mg mL^–1^ is required to ensure the absence of aggregates of polymer 9/1-Oct-Cat **6a** during the adsorption step. The high count rate of polymer 1/1-FOct-Cat **5b** is considered an outlier because of five data points at higher concentration with low count rates around the reference, which indicate no aggregation at much higher concentrations. As a result from the suppression of aggregation in the monolayer formation process, the wettability of the monolayers turned out to depend to a larger extent on the substrate than for the standard coatings (Figure 6). The monolayer does not lead to a significant change of the WCAs on PMMA, but the nonfluorinated monolayers **5a** and **6a** cause a drop of the WCA from (94 ± 8)° to (69 ± 7)° on PS. Noteworthy, the monolayer with the lowest WCA (73 ± 17)° on PTFE is formed by polymer 1/1-FDec-Cat **5c**, which contains the longest fluoroalkyl groups. However, except for 1/1-Oct-Cat **5a** all polymers lead to a reduction of the WCA of PTFE, indicating again successful adsorption that seems to be only minorly affected by fluorophilic groups at the coating polymer. SEM images confirm a smooth surface of the 1/1-FOct-Cat **5b** monolayer-coated substrates (Figures 5, S41, and S42).

## Conclusions

PTFE, PS, and PMMA were coated with six different polyglycerol-based coatings with varying hydrophobic/fluorophilic units and varying lengths of the hydrophilic block. SEM and DLS analyses indicate the formation of aggregates during the coating process. The main conclusions from the measurements of the WCAs of the coatings are (i) *a longer hydrophilic segment (polymers***6a***–***c***) results in hydrophilic coatings with a WCA of about 60°,* (ii) *a longer fluorocarbon chain on polymers***5c***and***6c***vs***5b***and***6b***does not increase the hydrophobicity or fluorophilicity of the coatings noticeably,* (iii) *the wettability of the coatings is only minorly dependent on the substrate (PTFE, PS, PMMA), showing the versatility of the coatings*, (iv) *no clear effect of fluorophilic adsorption of fluoroalkylated coating polymers on PTFE.* However, the low WCAs for most of the highly fluorinated substrate coating systems, like 9/1-FDec-Cat **6c** (61 ± 24)° or monolayered 1/1-FDec-Cat **5c** (73 ± 17)° on PTFE might indicate a potential fluorophilic interaction between the fluorinated coating and substrate that prevents fluoroalkyl groups from orienting toward the aqueous phase. Especially, in the case of PTFE surfaces the coatings have shown significantly reduced WCAs even below 65° to enable a potential use in anti-biofouling applications according to Berg’s law.^43−45^

## Supporting Information Available

NMR spectra, GPC chromatograms, SEM images, dissipation plot, pictures of coatings, educt and product masses, experimental details of GPC, and ellipsometry (PDF)
