# Supplementary material for: Effect of Fluorophilic- and Hydrophobic-Modified Polyglycerol-Based Coatings on the Wettability of Low Surface Energy Polymers
Source: Langmuir. 2025 Jan 27;41(5):3305–14. doi: 10.1021/acs.langmuir.4c04220 (PMC11823634; doi:10.1021/acs.langmuir.4c04220)
Supplement: Supplementary file 1 — la4c04220_si_001.pdf [file la4c04220_si_001.pdf]

Effect of Fluorophilic- and Hydrophobic-Modified Polyglycerol-Based Coatings on the Wettability of Low Surface Energy Polymers

*Florian Junge and Rainer Haag**

Institut für Chemie und Biochemie, Freie Universität Berlin, Takustr. 3, 14195 Berlin, Germany

E-mail: haag@chemie.fu-berlin.de

Table of Contents

Ellipsometry 2
Gel Permeation Chromatography (GPC) 3
Synthesis-Related Data 3
Pictures of Coatings 1 27
Dissipation Plots 28
Pictures of Coatings 2 28
Distributions of Hydrodynamic Diameters from DLS 30
SEM Images 32

Ellipsometry

The thickness of a coating of 9/1-Oct-Cat **6a** on PS coated titanium QCM-D sensors (QSX 310, Biolin Scientific, Gothenburg, Sweden) was determined by ellipsometry using a SENpro ellipsometer SE 800 from Sentech (Berlin, Germany) in psi,delta-mode in a spectral range of 370 nm – 1050 nm with a goniometer angle of 70°. Three titanium sensors were cleaned with ethanol and acetone and coated with 30 µL of a 1 w% polystyrene (from Falcon petri dish 351008) in toluene using a WS-650MZ-23NPPB spin coater from Laurell (Lansdale, USA) which was operated with a nitrogen pressure of 4 bar and a 0523-101Q-G588DX vacuum pump from Gast (Redditch, UK). The homogenicity of the PS layer was confirmed using ellipsometry and contact angle measurement. The thickness of each layer of each sensor was measured at five different positions. The thickness and the refractive index of the top most layer was interpolated with the built-in fit function of the SpectraRay/3 programm using 500 iterations. The modulation of the layers assumed a oscillating titanium layer (all parameters except for "epsilon-infinity-imag" were interpolated together with the TiO_2 thickness by measuring the uncoated titanium sensors), followed by TiO_2 , the polystyrene cauchy layer, lPG copolymer cauchy layer and air (n = 1.00).

The PS coating thicknesses of the sensors were: (45.7 ± 0.3) nm, (48.7 ± 0.2) nm and (33.1 ± 0.1) nm and of the sensors that were later-on coated with 9/1-Oct-Cat **6a** (32.2 ± 0.3) nm, (32.6 ± 0.1) nm and (52.8 ± 0.2) nm. The thickness of the 9/1-Oct-Cat **6a** coating was (11.1 ± 0.3) nm, (8.1 ± 0.5) nm and (4.2 ± 0.4) nm respectively. WCA of PS coating on titanium sensors: (95 ± 2)°. WCA of 9/1-Oct-Cat **6a** coating on PS / titanium sensors: (78 ± 2)°.

Gel Permeation Chromatography (GPC)

GPC was performed using a PSS SECcurity system of the 1200 series from Agilent (Waldbronn, Germany) equipped with a PL gel column (particle size: 5 µm) from the same company. Polystyrene was used as calibration standard and ethylene glycol as internal standard. Tetrahydrofuran (THF) for HPLC was employed as the mobile phase, at a flow rate of 1 mL min^{-1}. A solution of the respective polymer in THF (50 µL, 6 mg ml^{-1}) was injected onto the column.

Synthesis-Related Data

Table S1 Weight of the educts and products of the polymerization.

polymer	Product [g]	Bromide [g]	EEGE [mL]	AGE [mL]	*i*Bu$_3$Al [mL]
1/1-copolymer **1**	5.01	7.80	19	15	39 + 13
9/1-copolymer **2**	8.55	1.11	24	2.2	5.5 + 1.9

Table S2 Weight of the educts and products of the thiol-ene coupling.

polymer	Educt [g]	Product [g]	Amine [g]	Thiol [mL]	DMPA[1] [µL]
1/1-Oct **3a**	0.751	0.660	1.21	0.924	205
1/1-FOct **3b**	0.746	1.76	1.21	1.24	204
1/1-FDec **3c**	0.767	0.929	1.38	1.55	210
9/1-Oct **4a**	1.51	0.805	0.580	0.445	98.6
9/1-FOct **4b**	1.50	0.956	0.581	0.599	98.4
9/1-FDec **4c**	1.41	1.41	0.586	0.735	98.9

[1] per addition

Table S3 Weight of the educts and products of the amide condensation.

polymer	Educt [g]	Product [g]	DCC [g]	Acid [g]
1/1-Oct-Cat **5a**	0.601	0.556	0.791	0.700
1/1-FOct-Cat **5b**	1.76	1.50	1.86	1.65
1/1-FDec-Cat **5c**	1.04[1]	0.573	1.01	0.889
9/1-Oct-Cat **6a**	0.619	0.693	0.283	0.246
9/1-FOct-Cat **6b**	0.941	0.768	0.395	0.350
9/1-FDec-Cat **6c**	1.40	0.505	0.566	0.502

[1] Higher mass than the product mass obtained in the previous step for an unknown reason.

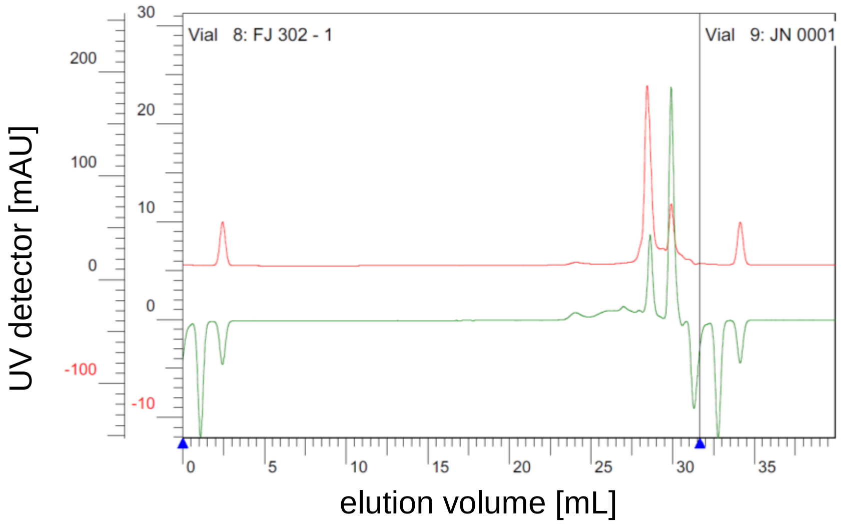

Figure S1. GPC chromatogram of polymer **1**.

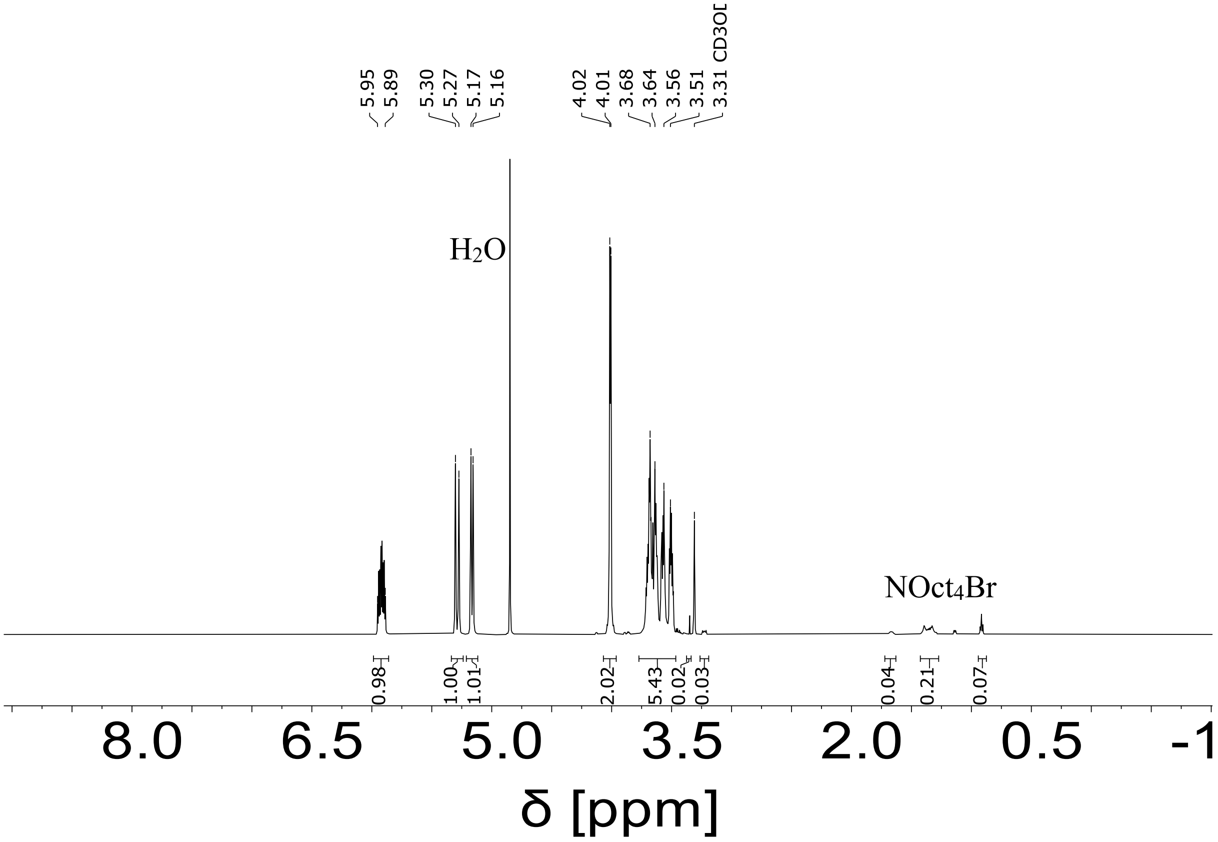

Figure S2. ^{1}H NMR spectrum (600 MHz, CD_3OD) of 1/1-copolymer **1**.

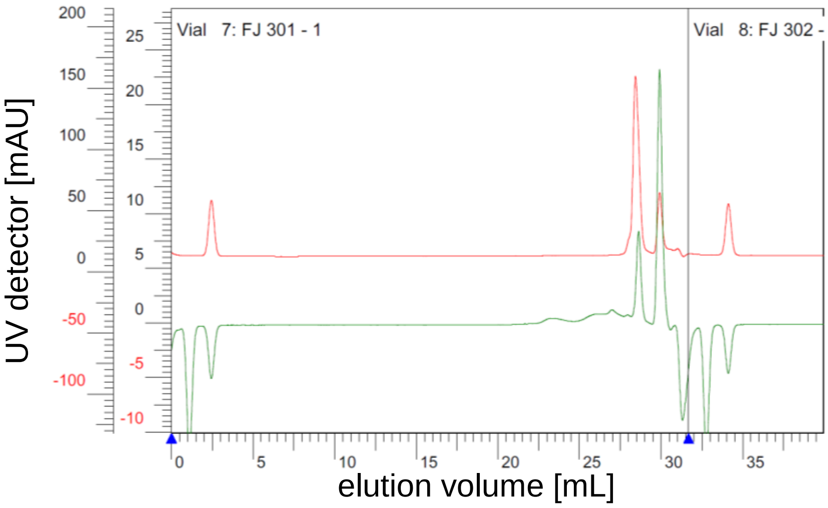

Figure S3. GPC chromatogram of polymer **2**.

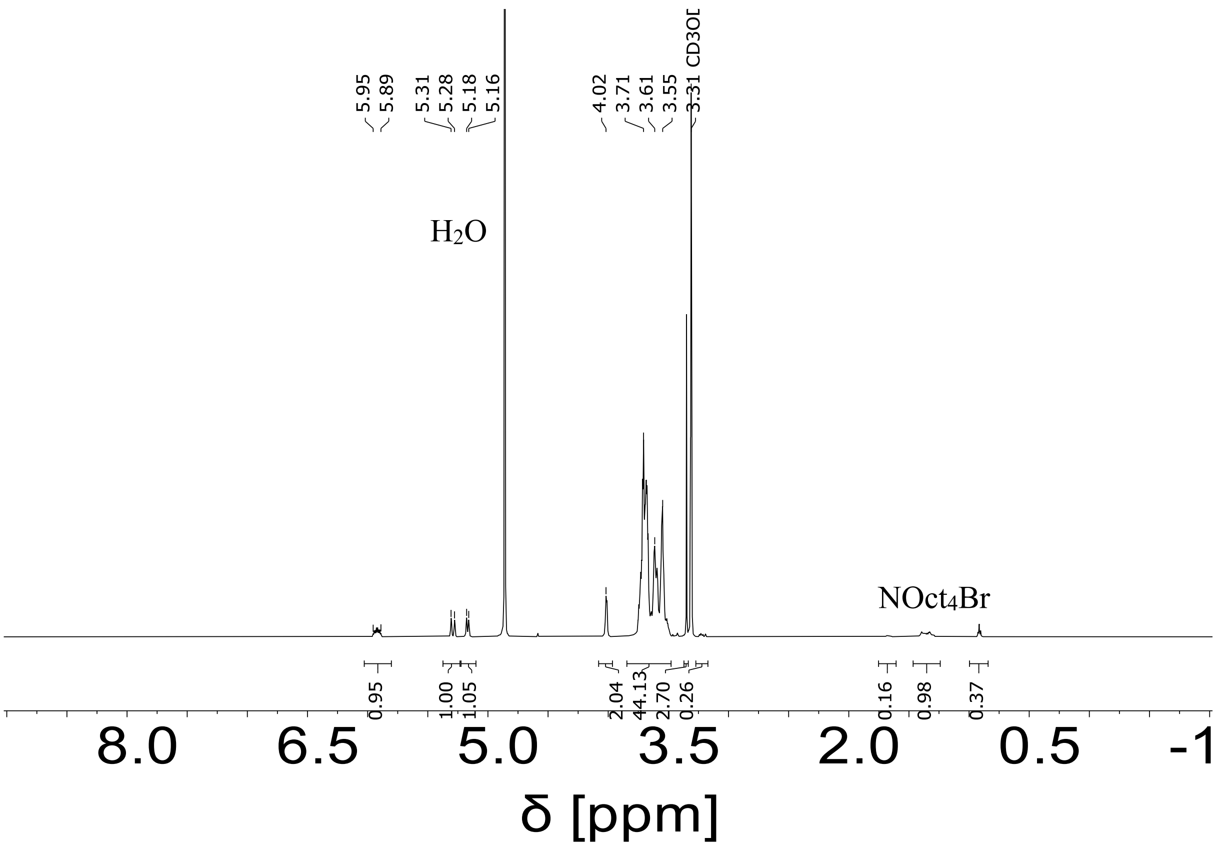

Figure S4. ^{1}H NMR spectrum (600 MHz, CD_3OD) of 9/1-copolymer **2**.

Table S4. Degree of functionalization of the intermediates determined by NMR.

**polymer**	**Amine [%]**	**(Fluoro)alkyl [%]**
1/1-Oct **3a**	67	33
1/1-FOct **3b**	69	31
1/1-FDec **3c**	71	29
9/1-Oct **4a**	60	40
9/1-FOct **4b**	64	36
9/1-FDec **4c**	57	43

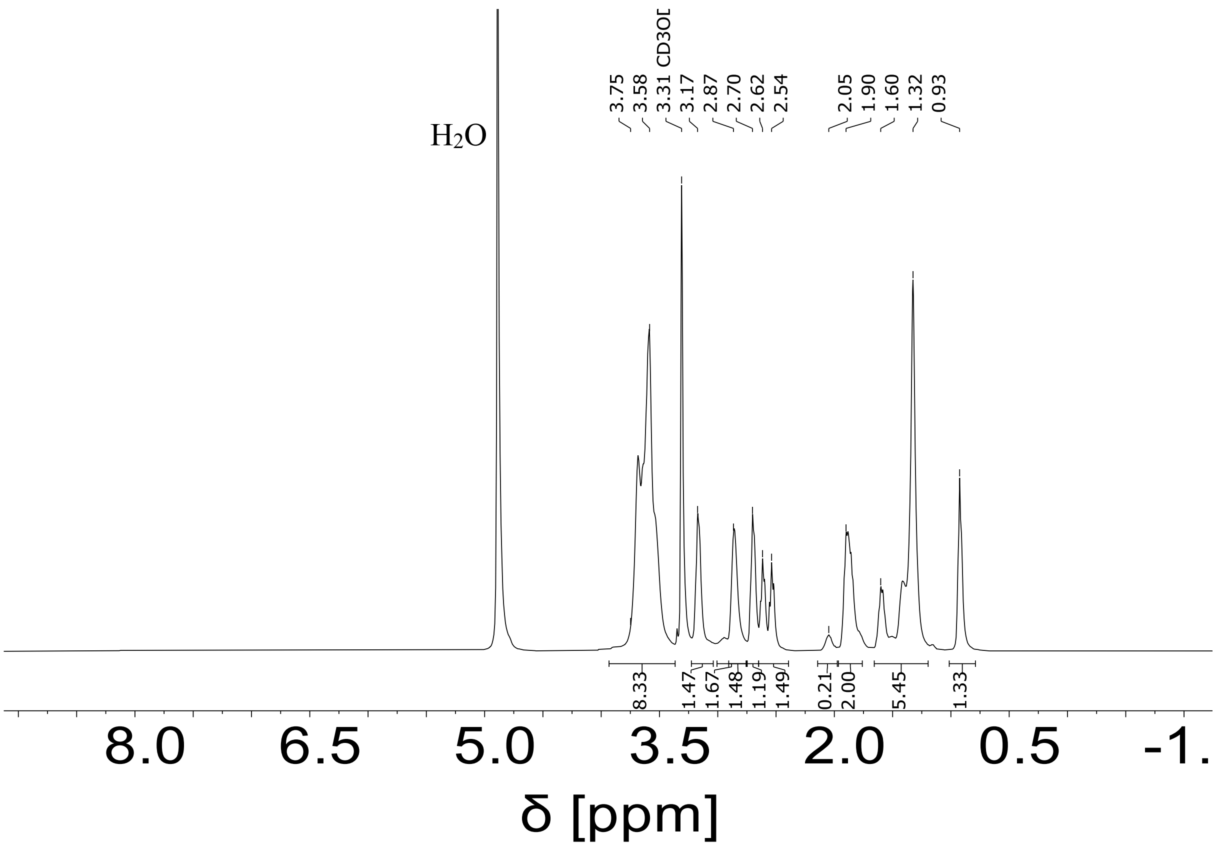

Figure S5. ^{1}H NMR spectrum (400 MHz, CD_3OD) of 1/1-Oct **3a**.

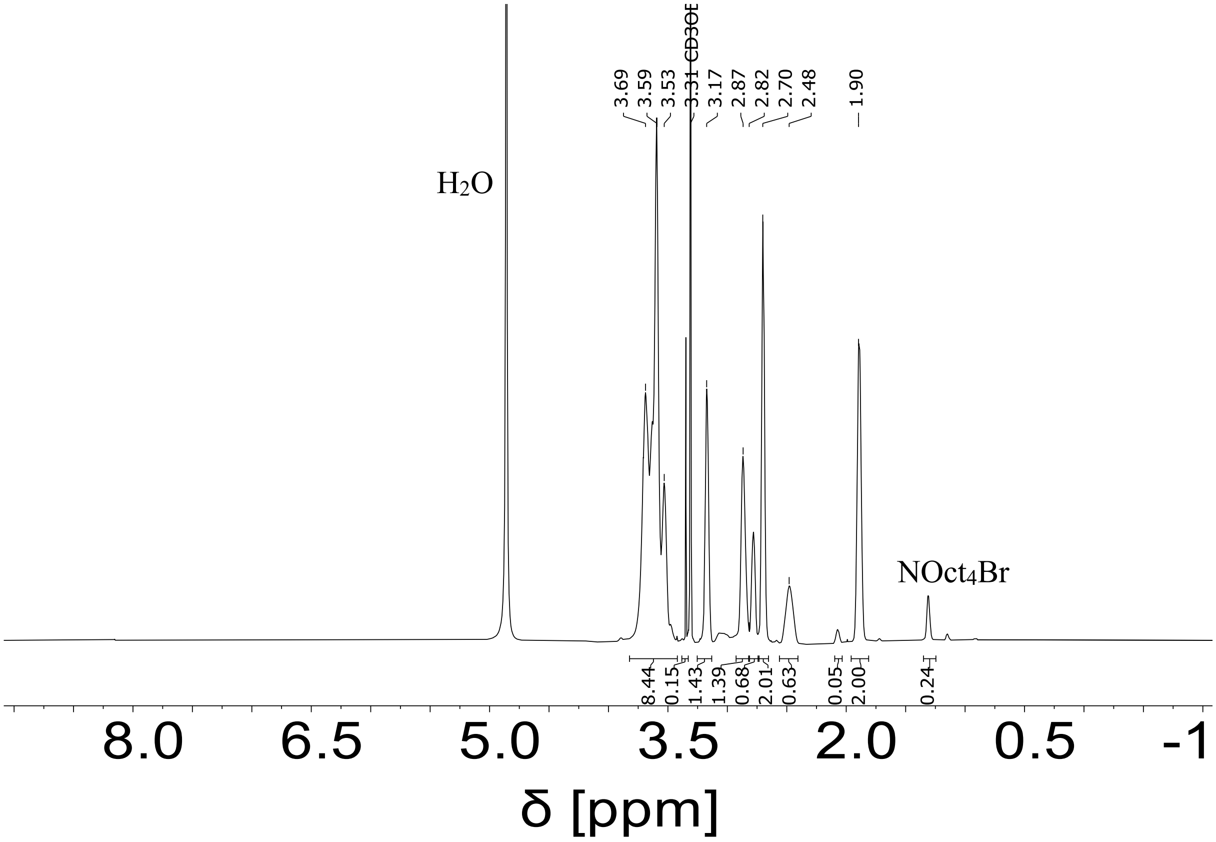

Figure S6. ^{1}H NMR spectrum (600 MHz, CD_3OD) of 1/1-FOct **3b**.

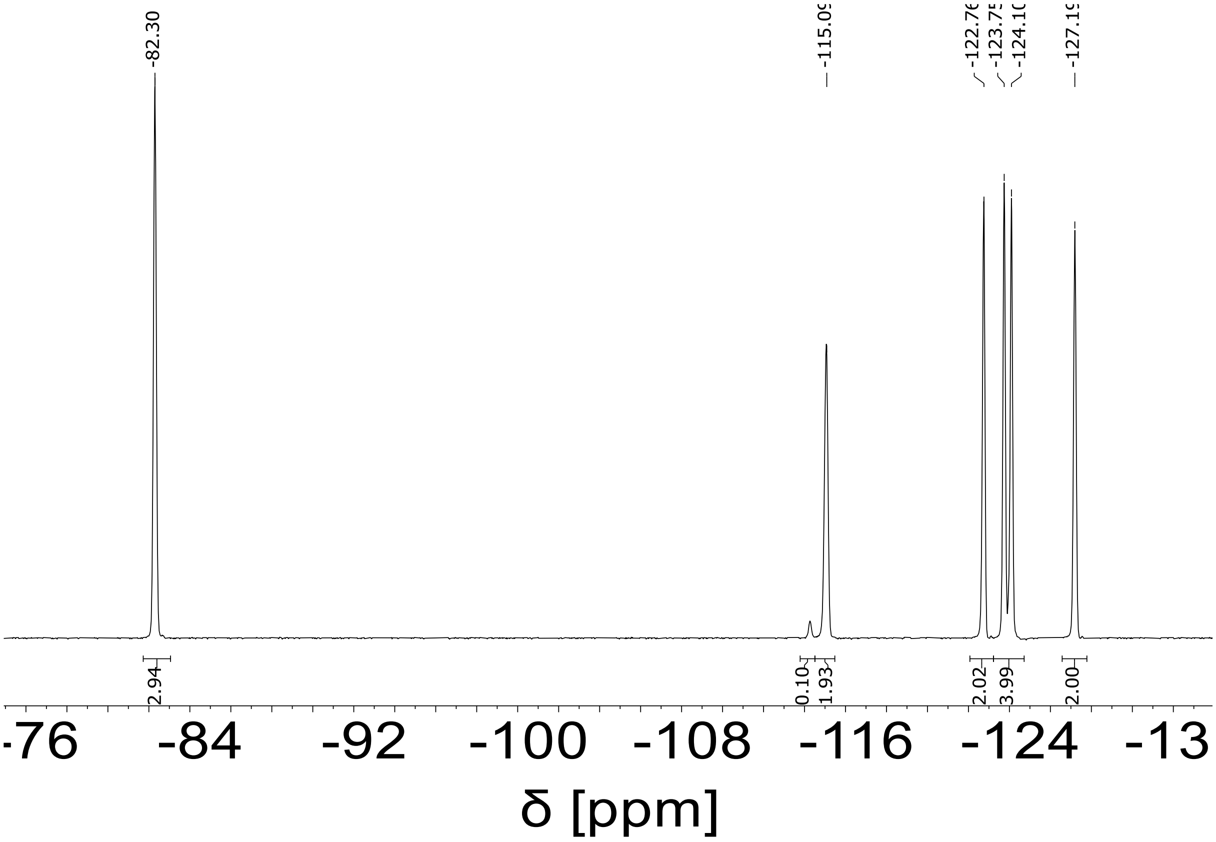

Figure S7. ^{19}F NMR spectrum (565 MHz, CD_3OD) of 1/1-FOct **3b**.

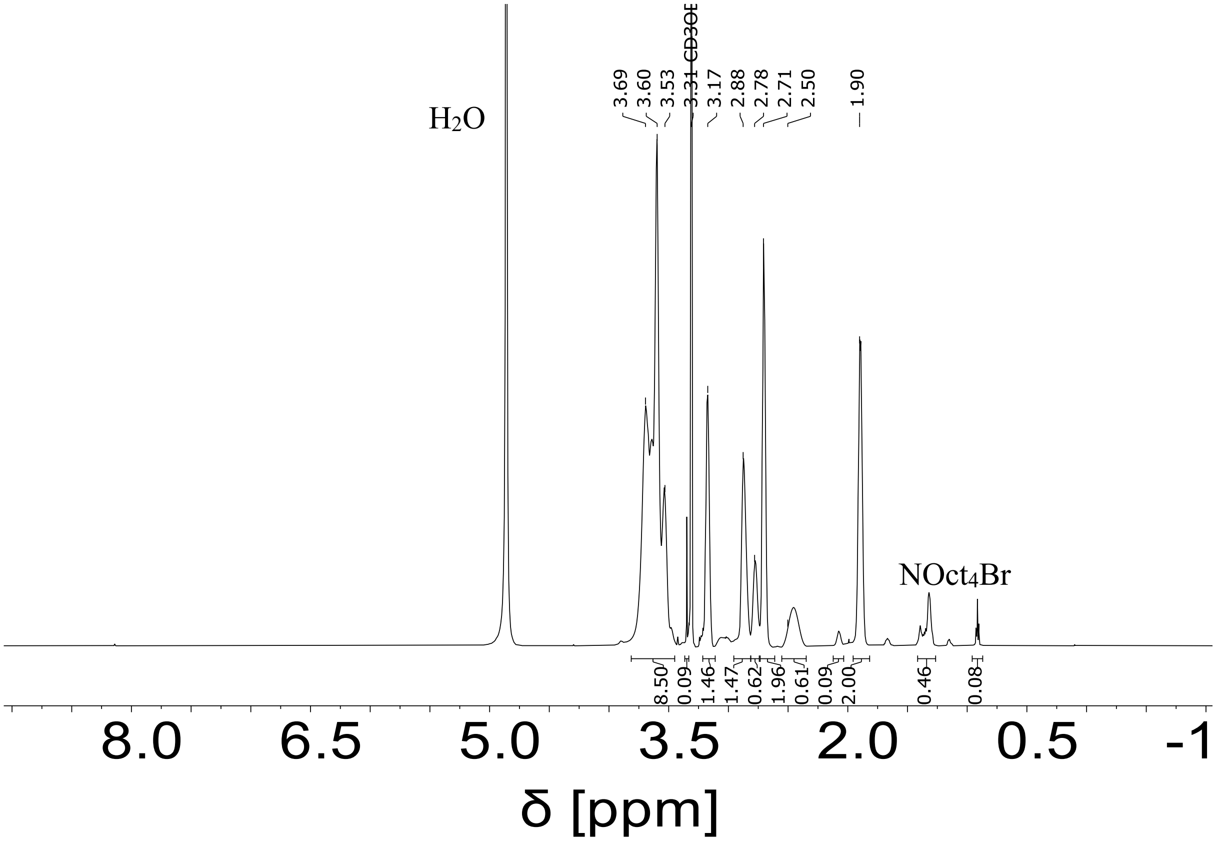

Figure S8. ^{1}H NMR spectrum (600 MHz, CD_3OD) of 1/1-FDec **3c**.

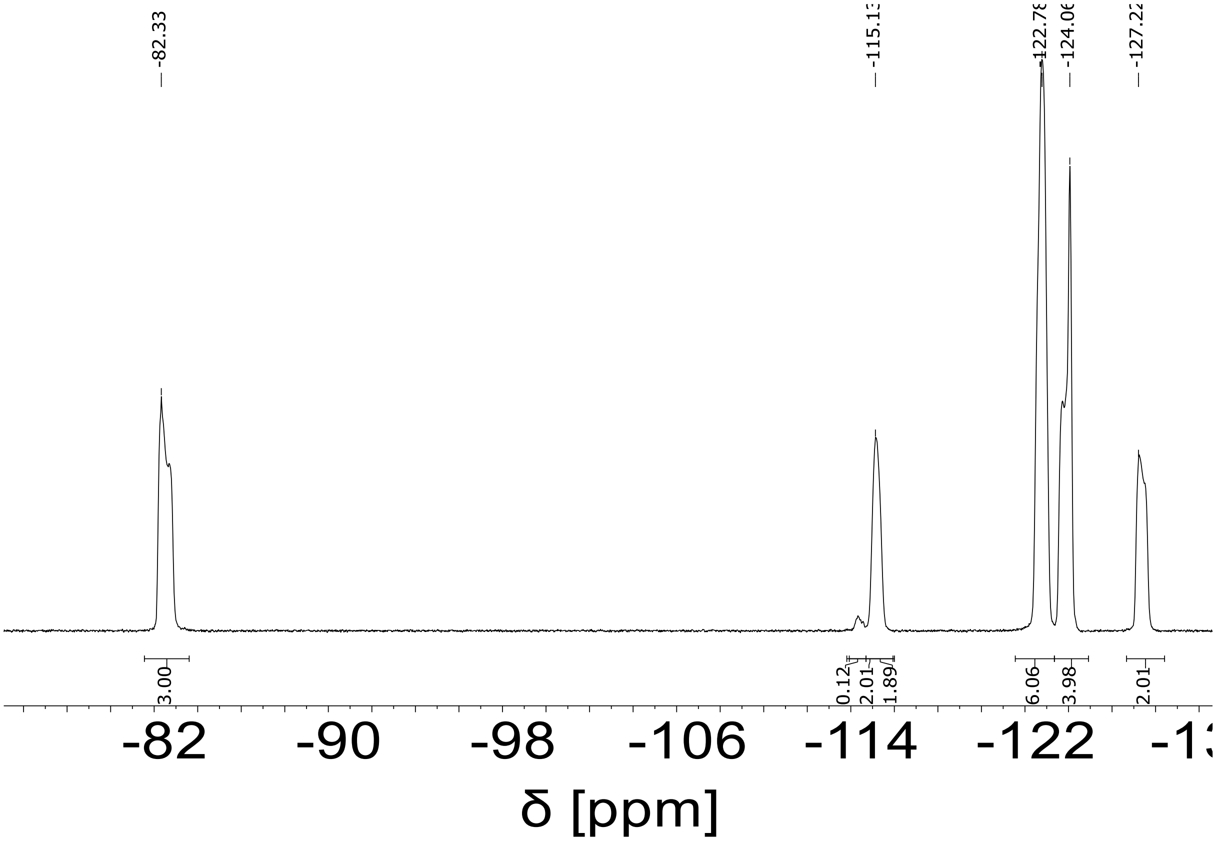

Figure S9. ^{19}F NMR spectrum (565 MHz, CD_3OD) of 1/1-FDec **3c**.

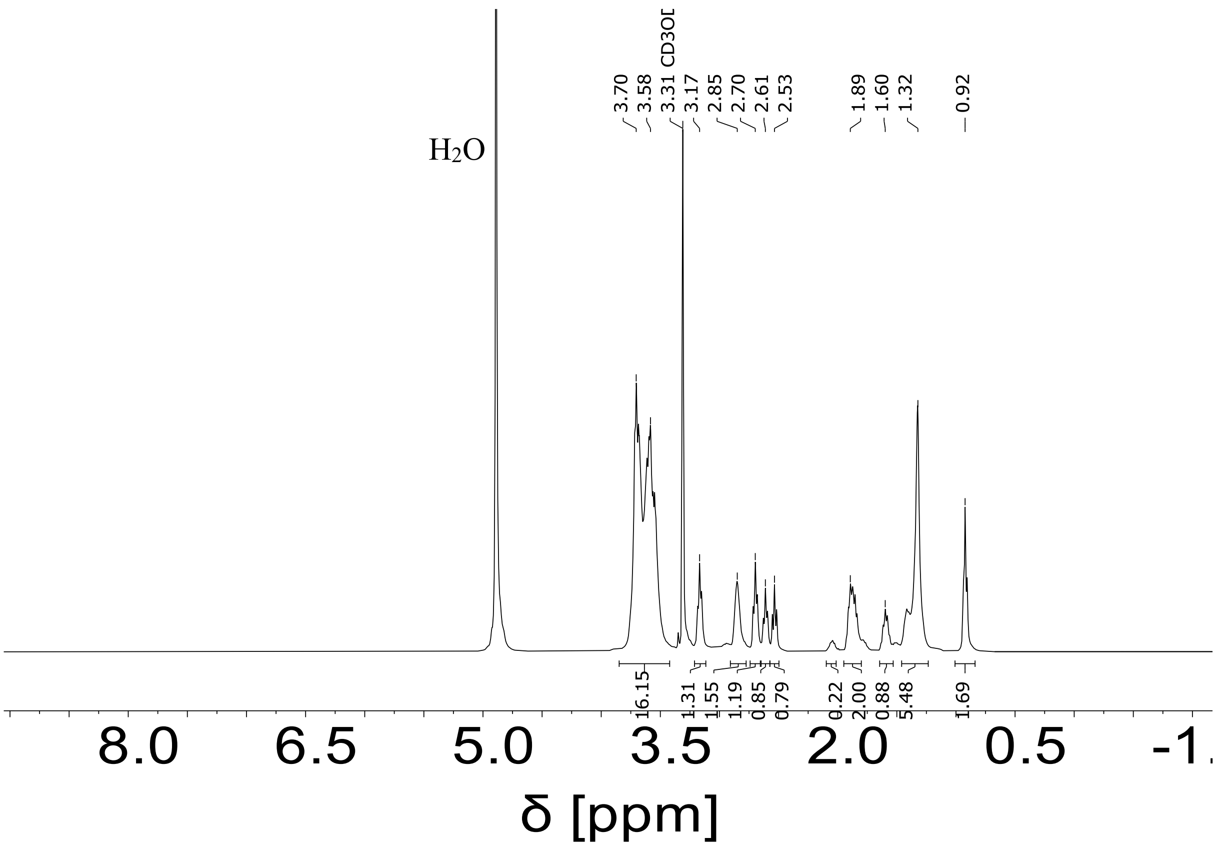

Figure S10. ^{1}H NMR spectrum (400 MHz, CD_3OD) of 9/1-Oct **4a**.

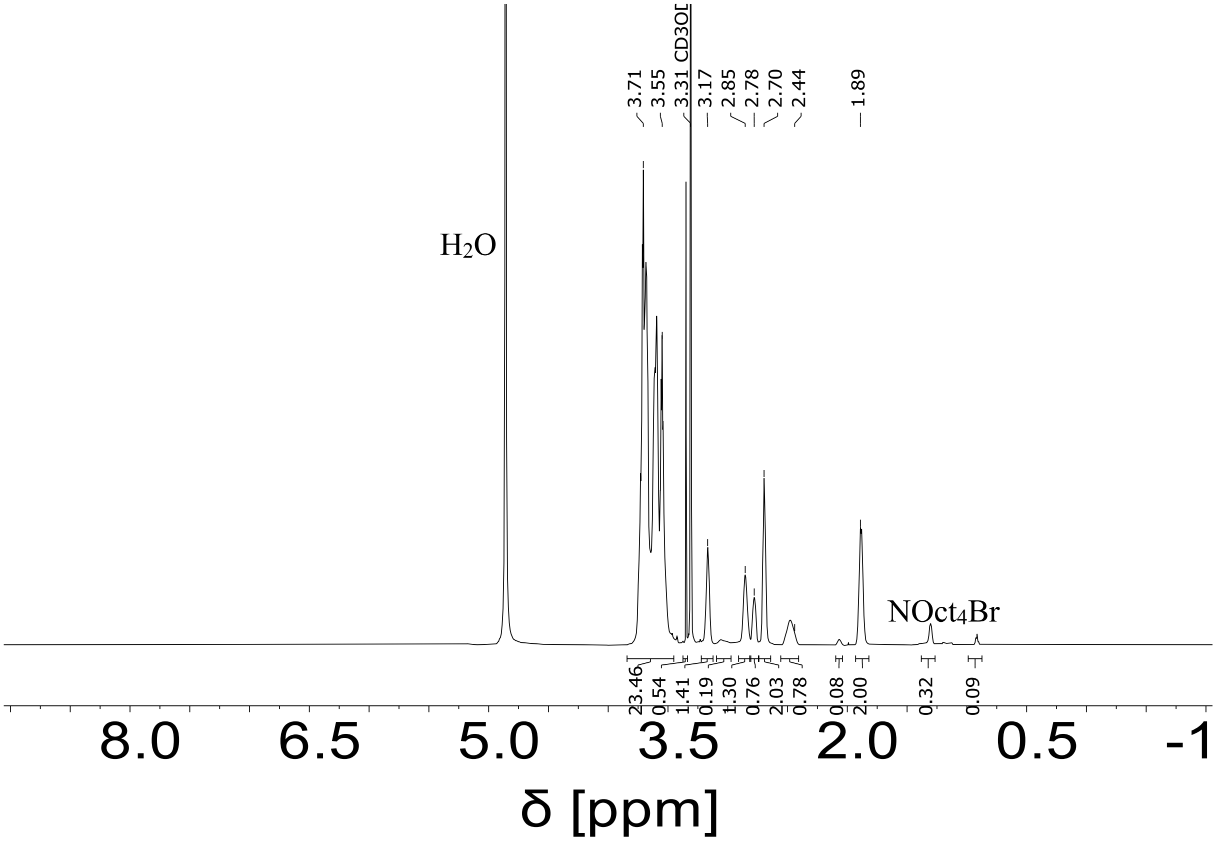

Figure S11. ^{1}H NMR spectrum (600 MHz, CD_3OD) of 9/1-FOct **4b**.

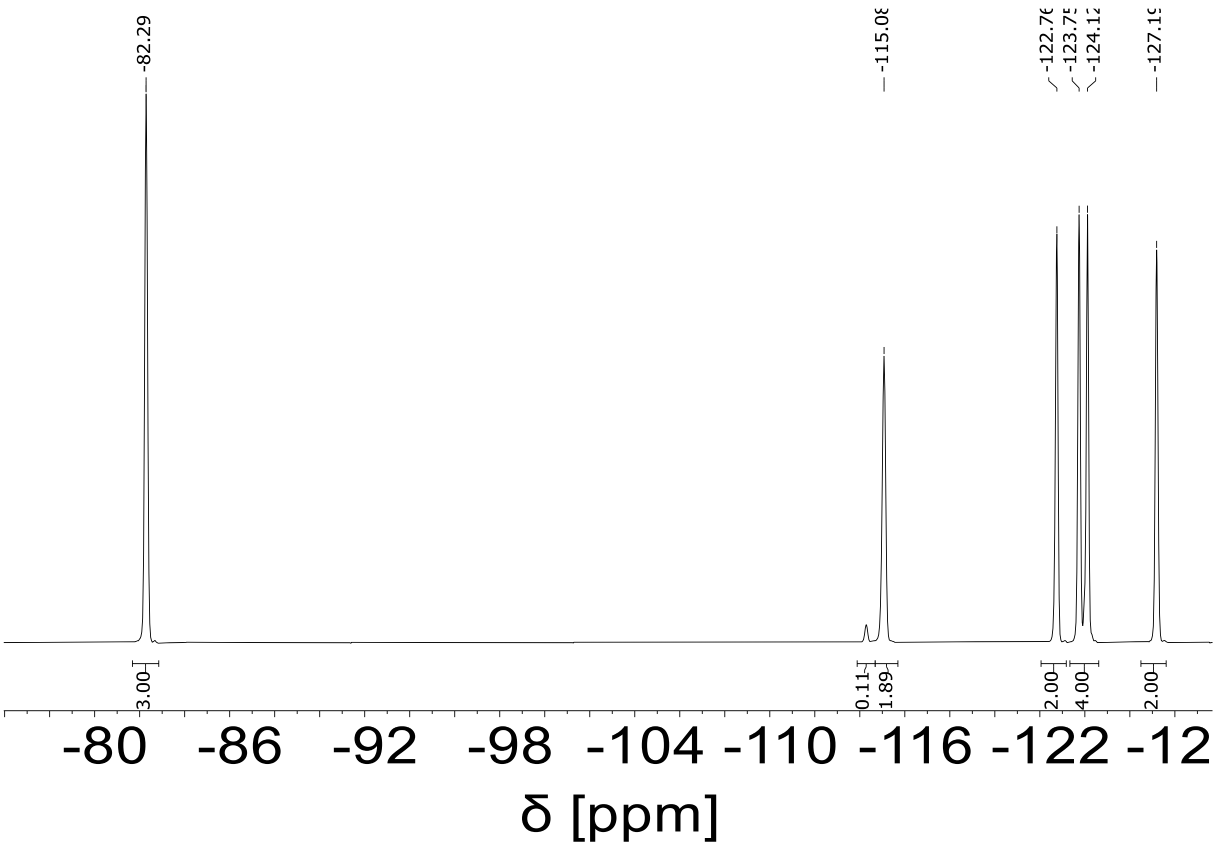

Figure S12. ^{19}F NMR spectrum (565 MHz, CD_3OD) of 9/1-FOct **4b**.

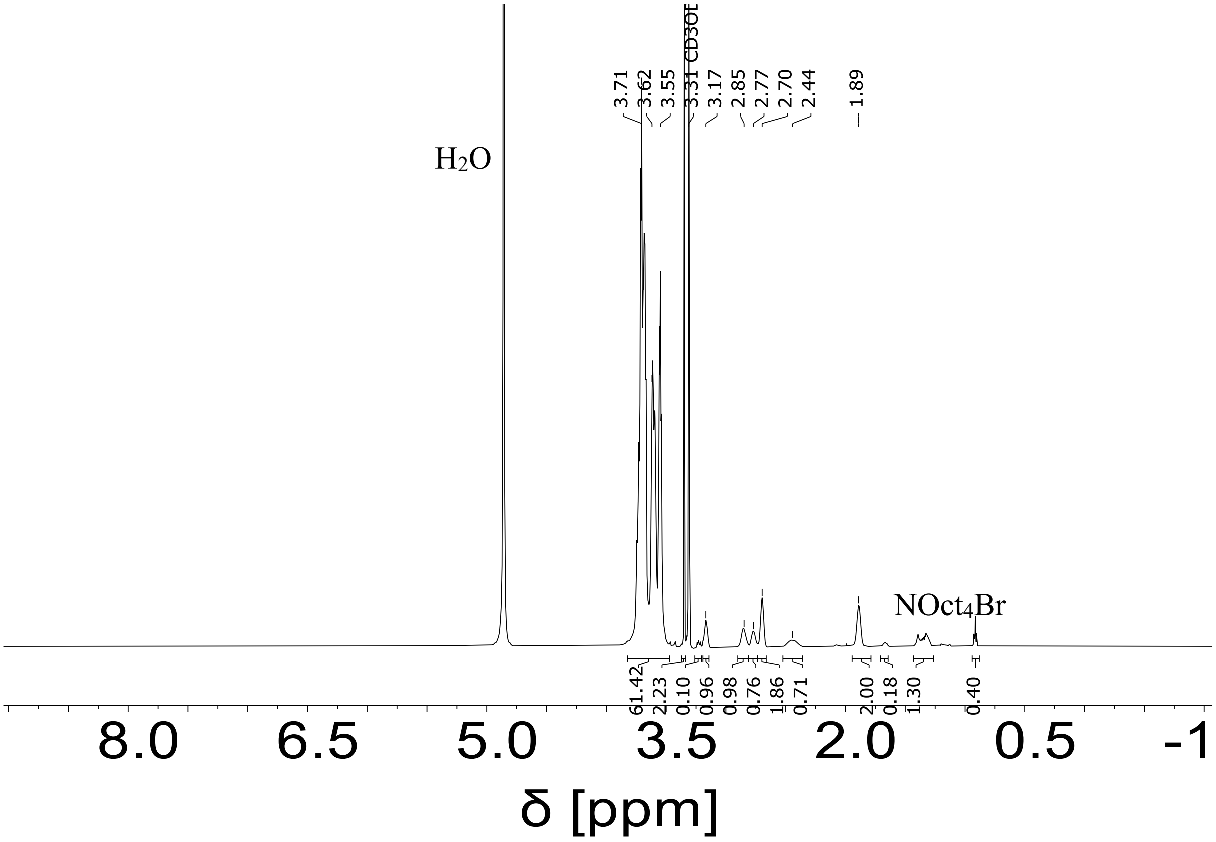

Figure S13. ^{1}H NMR spectrum (600 MHz, CD_3OD) of 9/1-FDec **4c**.

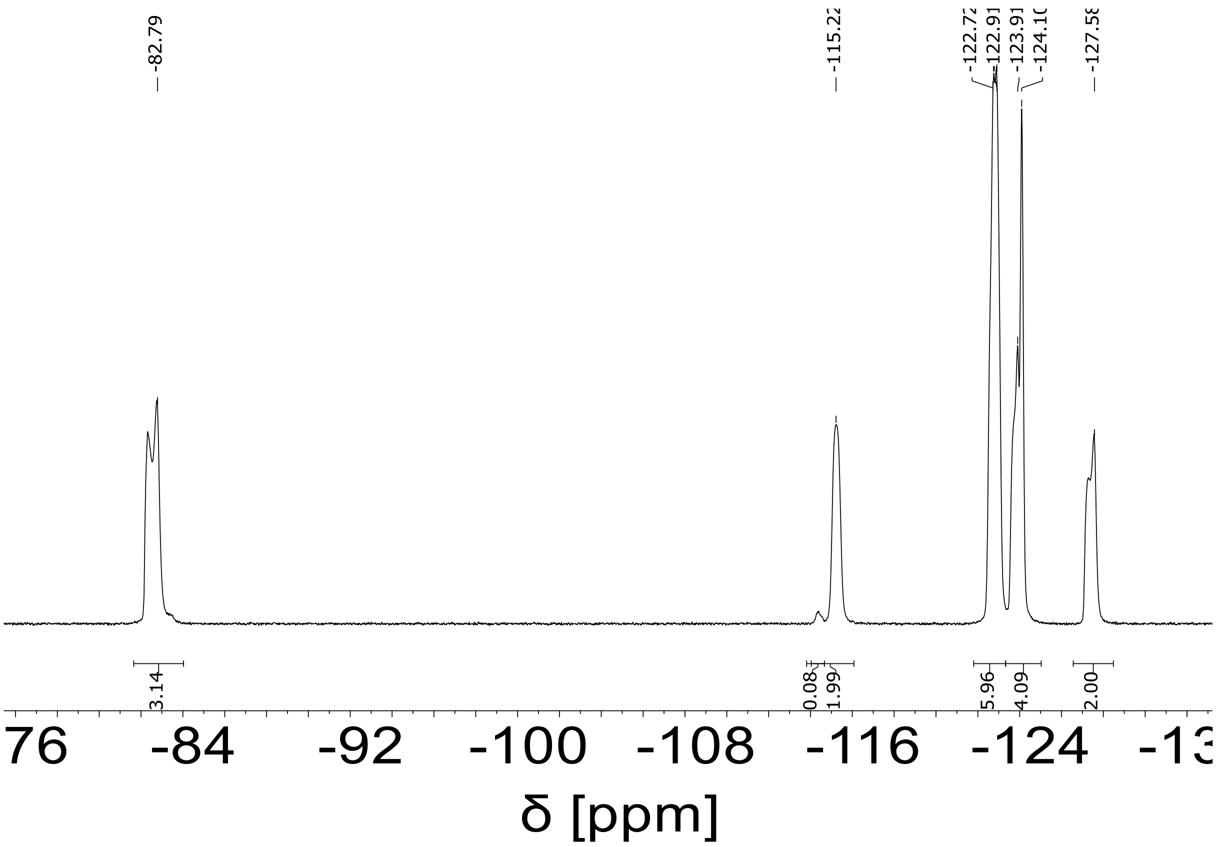

Figure S14. ^{19}F NMR spectrum (565 MHz, CD_3OD) of 9/1-FDec **4c**.

Table S5. Degree of functionalization of the final coating polymers determined by NMR.

polymer	Catechol [%]	Amine [%]	(Fluoro)alkyl [%]
1/1-Oct-Cat **5a**	36	27	36
1/1-FOct-Cat **5b**	39	29	31
1/1-FDec-Cat **5c**	39	37	24
9/1-Oct-Cat **6a**	20	40	41
9/1-FOct-Cat **6b**	27	36	36
9/1-FDec-Cat **6c**	56	16	28

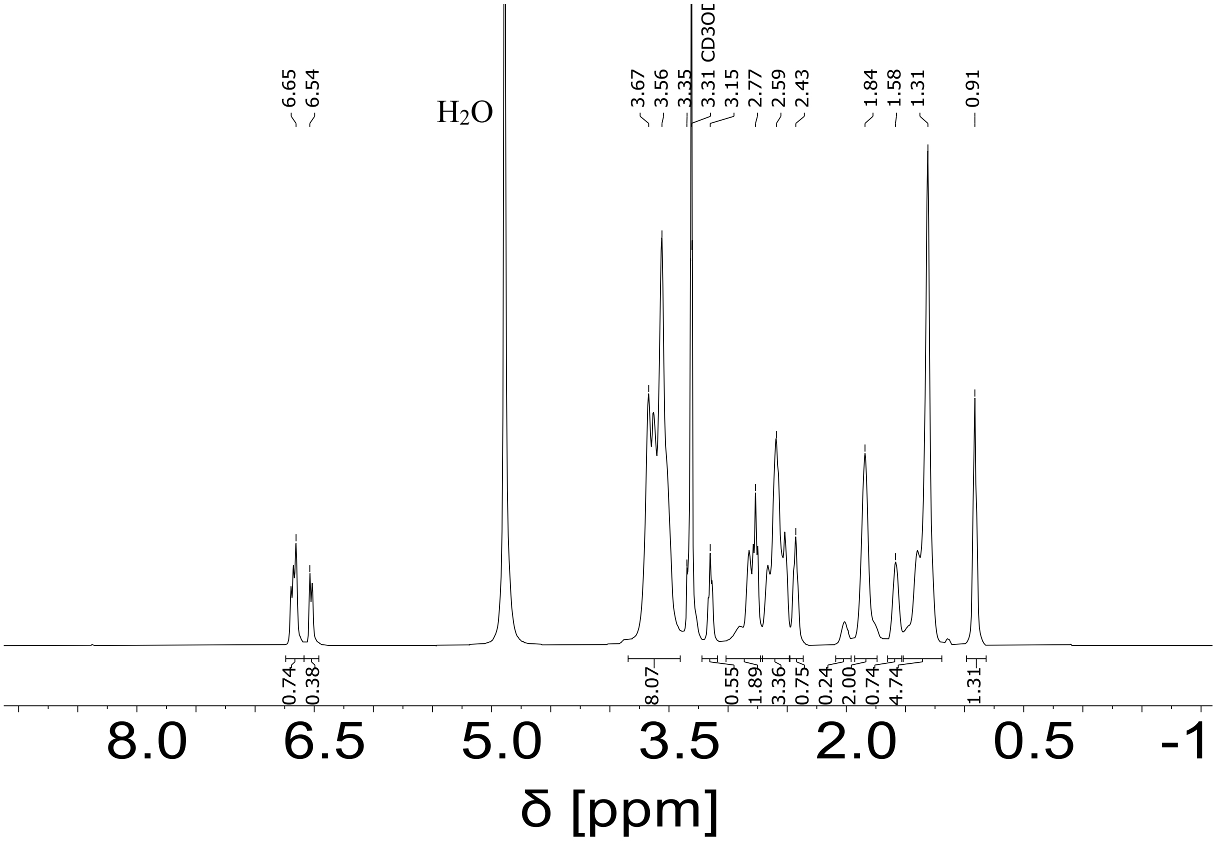

Figure S15. ^{1}H NMR spectrum (400 MHz, CD_3OD) of 1/1-Oct-Cat **5a**.

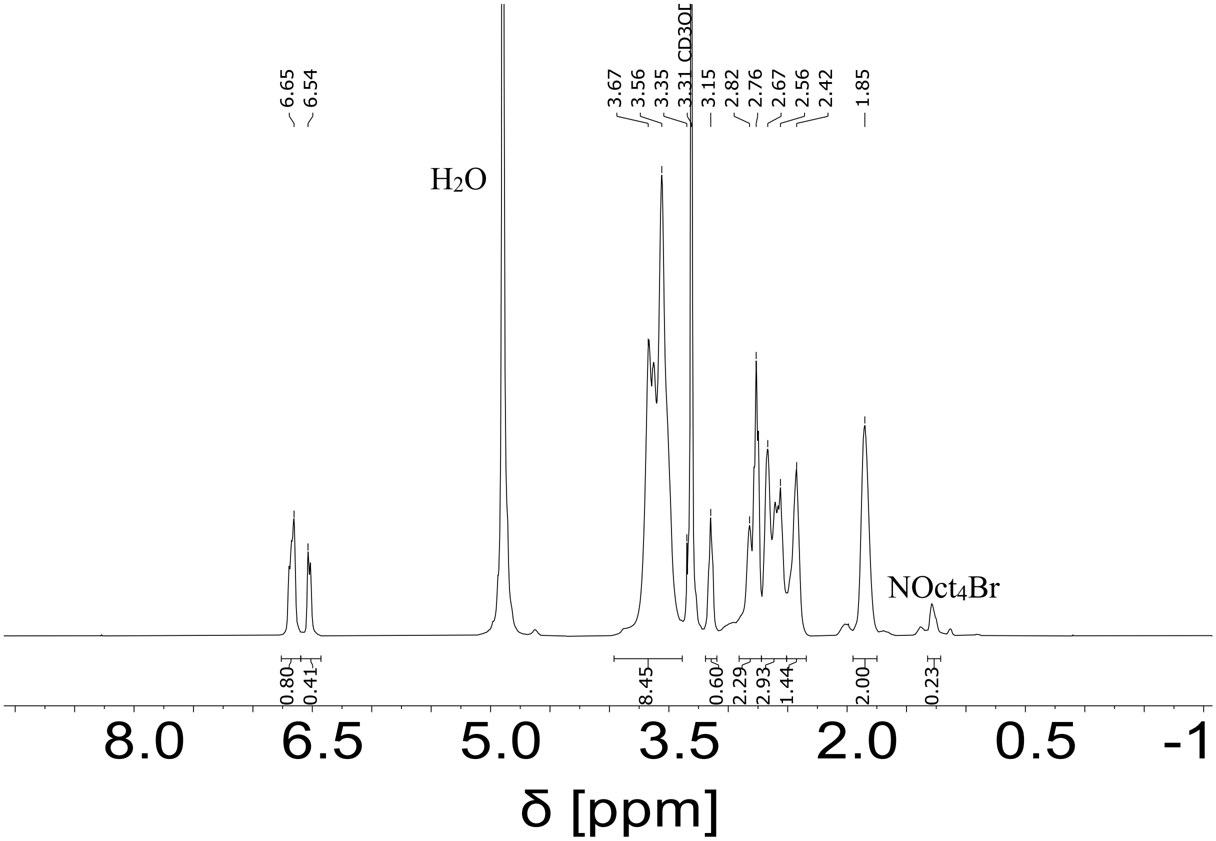

Figure S16. ^{1}H NMR spectrum (400 MHz, CD_3OD) of 1/1-FOct-Cat **5b**.

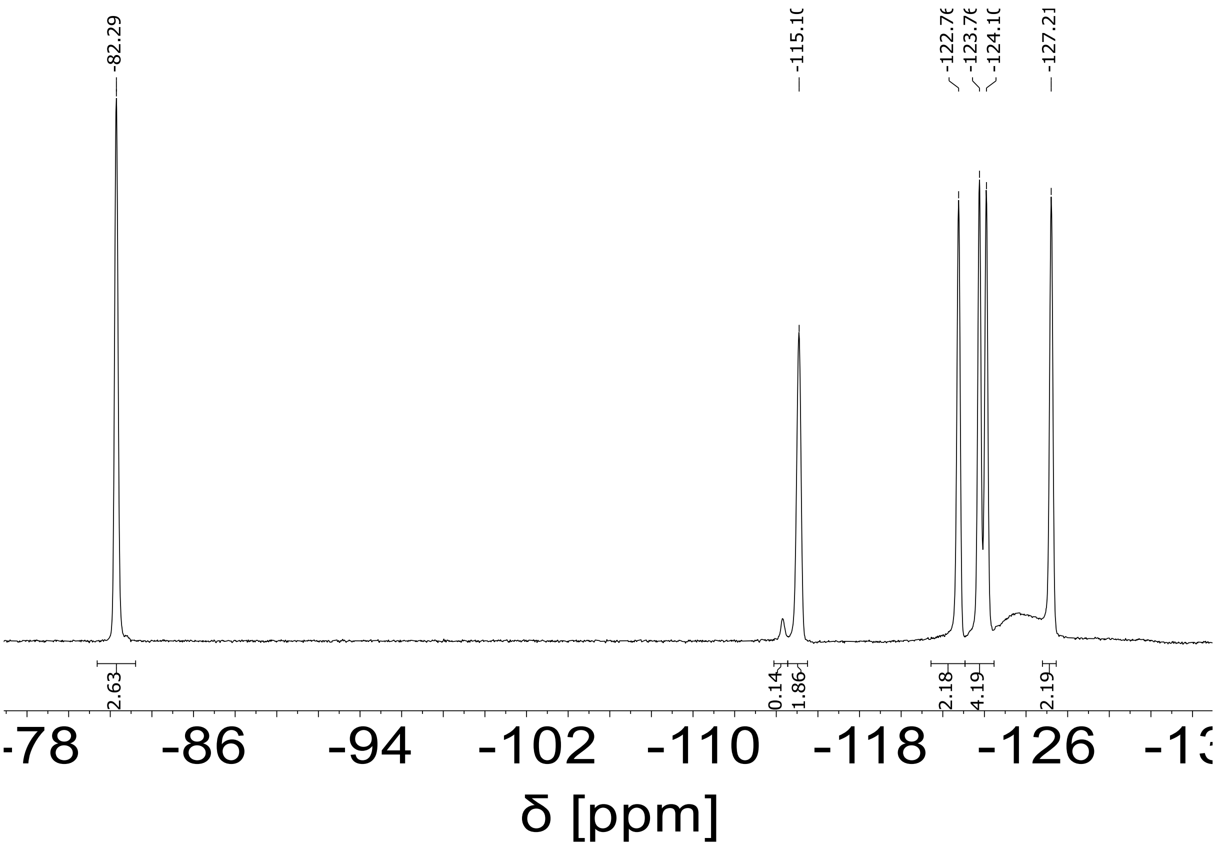

Figure S17. ^{19}F NMR spectrum (376 MHz, CD_3OD) of 1/1-FOct-Cat **5b**.

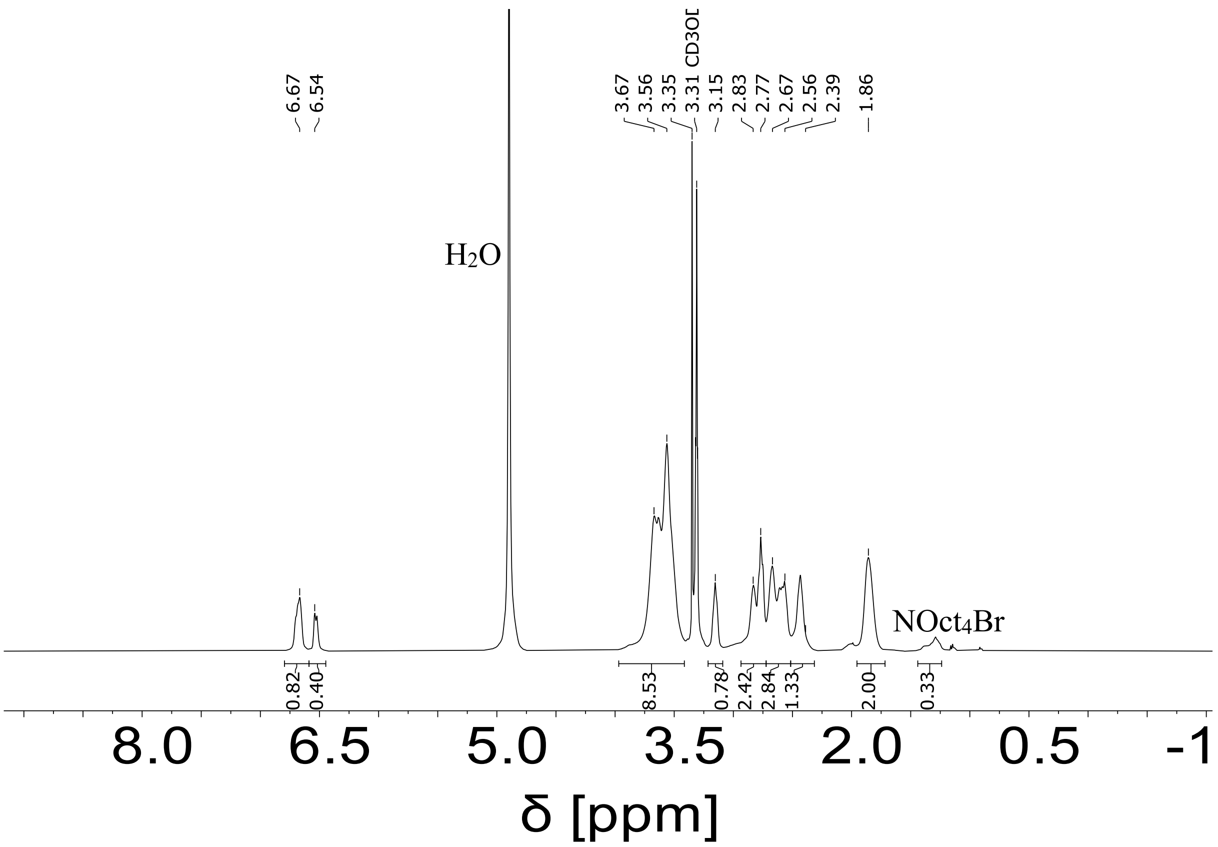

Figure S18. ^{1}H NMR spectrum (400 MHz, CD_3OD) of 1/1-FDec-Cat **5c**.

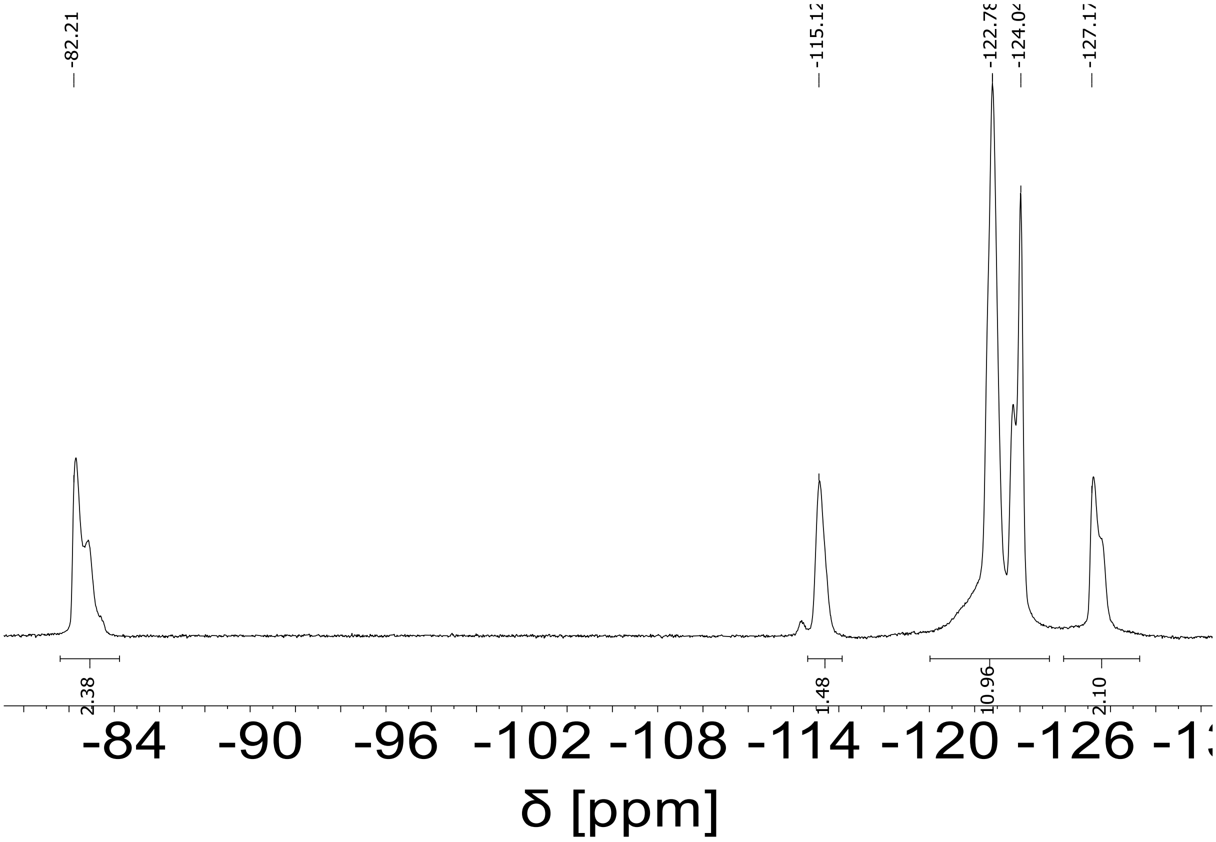

Figure S19. ^{19}F NMR spectrum (376 MHz, CD_3OD) of 1/1-FDec-Cat **5c**.

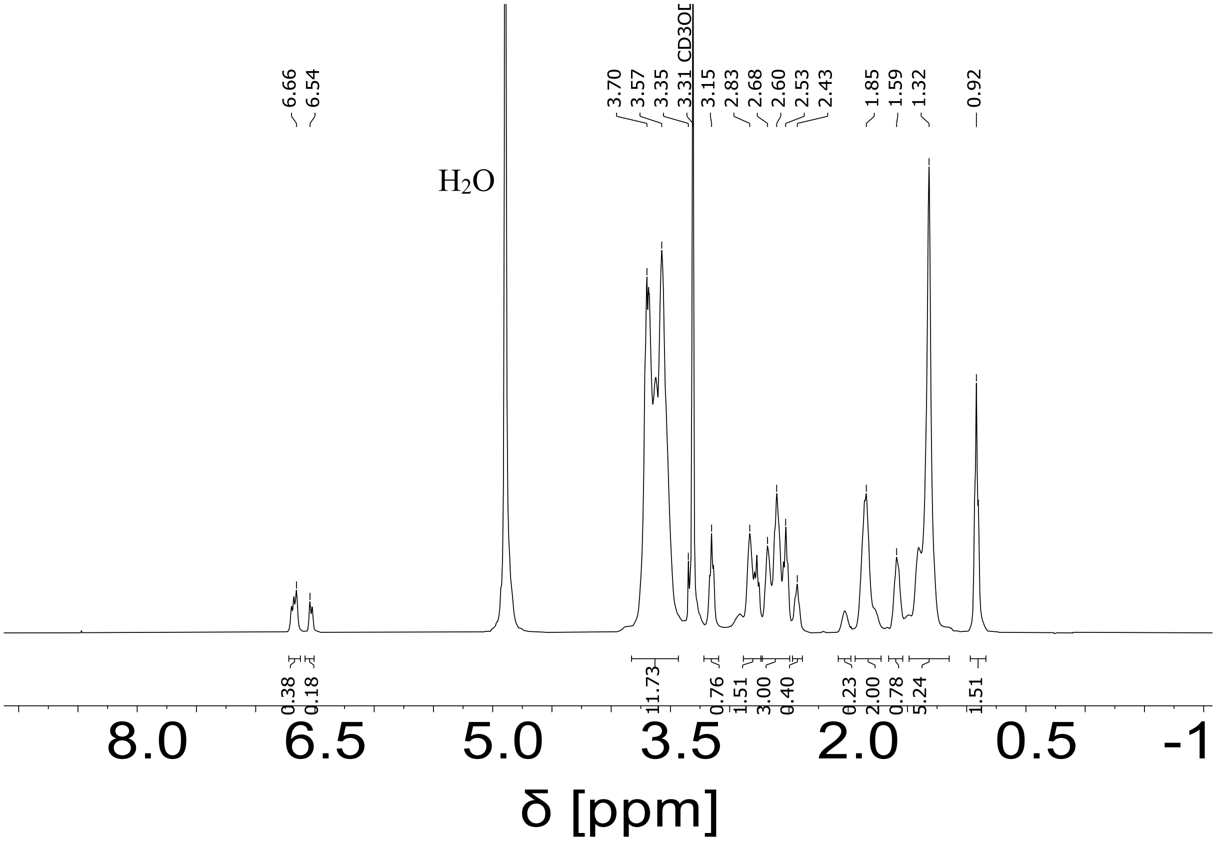

Figure S20. ^{1}H NMR spectrum (400 MHz, CD_3OD) of 9/1-Oct-Cat **6a**.

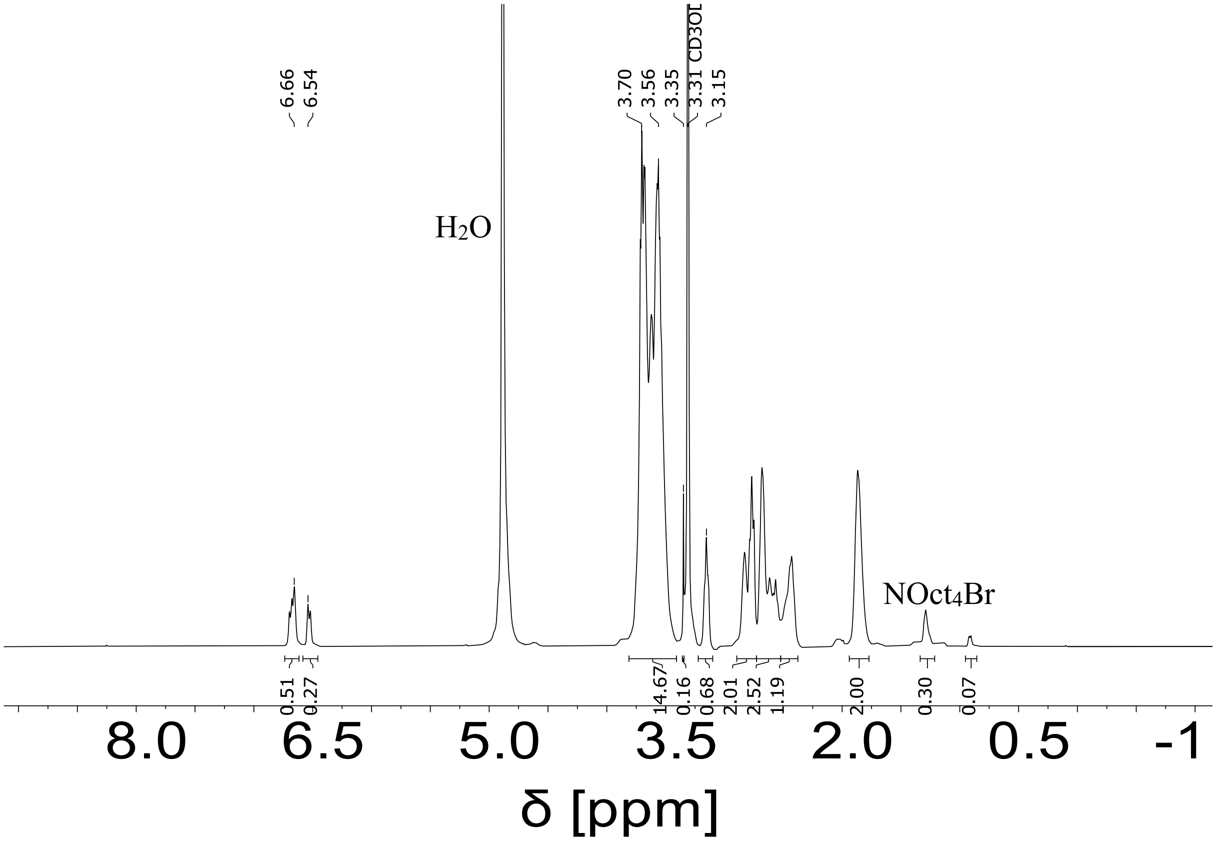

Figure S21. ^{1}H NMR spectrum (400 MHz, CD_3OD) of 9/1-FOct-Cat **6b**.

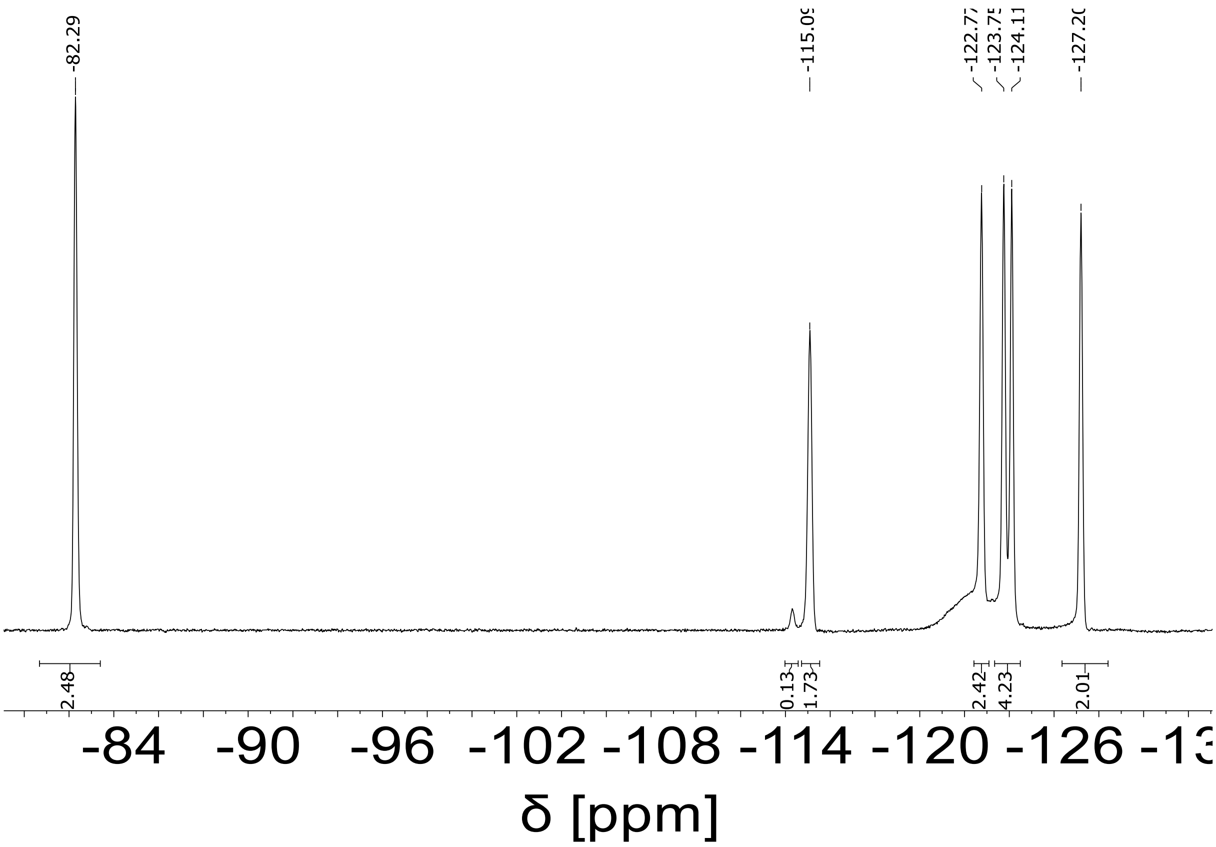

Figure S22. ^{19}F NMR spectrum (376 MHz, CD_3OD) of 9/1-FOct-Cat **6b**.

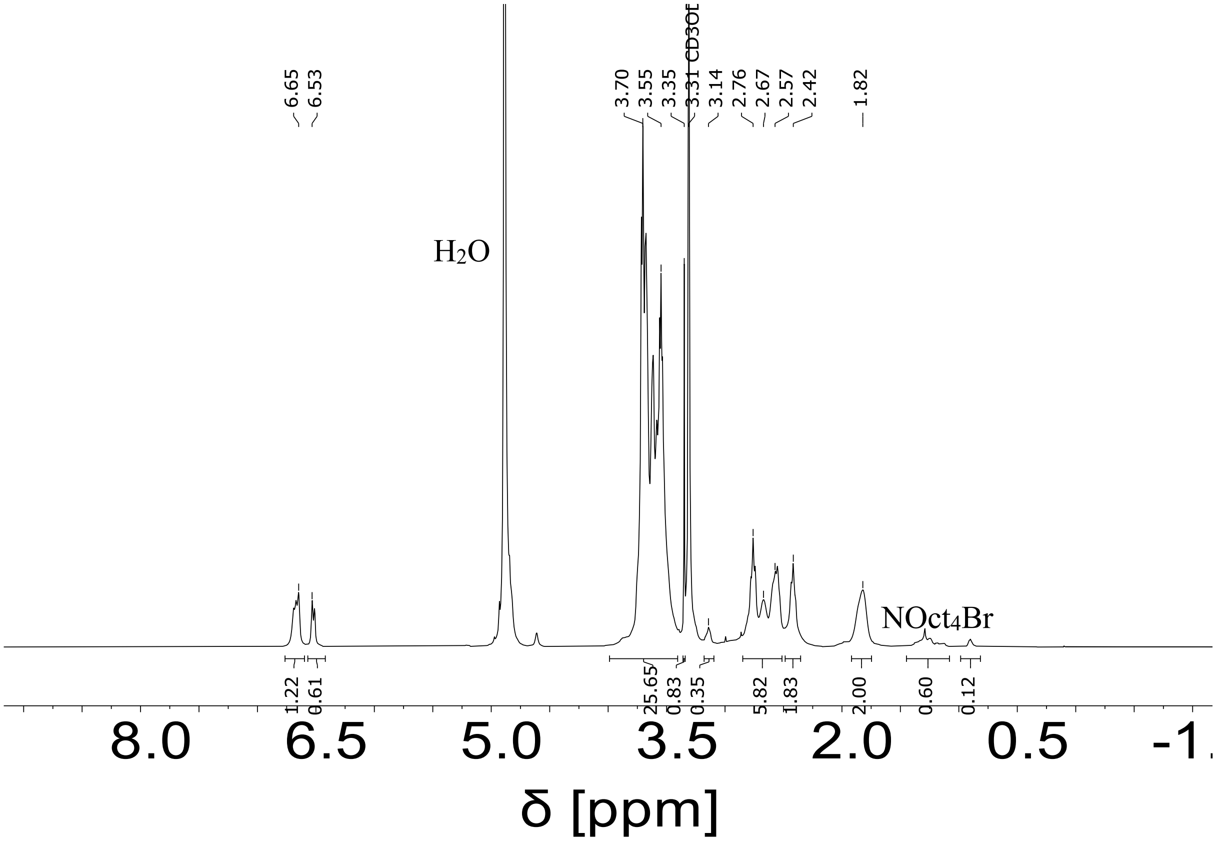

Figure S23. ^{1}H NMR spectrum (400 MHz, CD_3OD) of 9/1-FDec-Cat **6c**.

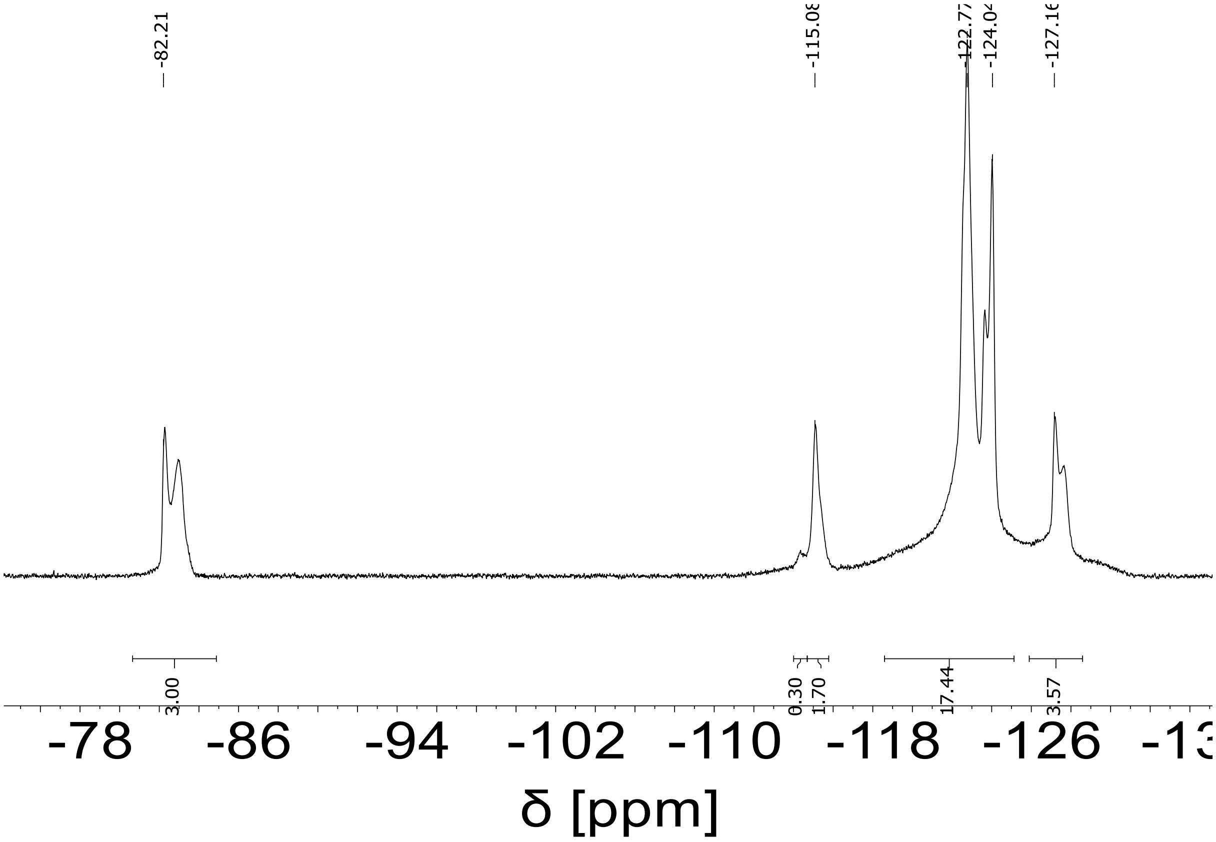

Figure S24. ^{19}F NMR spectrum (376 MHz, CD_3OD) of 9/1-FDec-Cat **6c**.

Pictures of Coatings 1

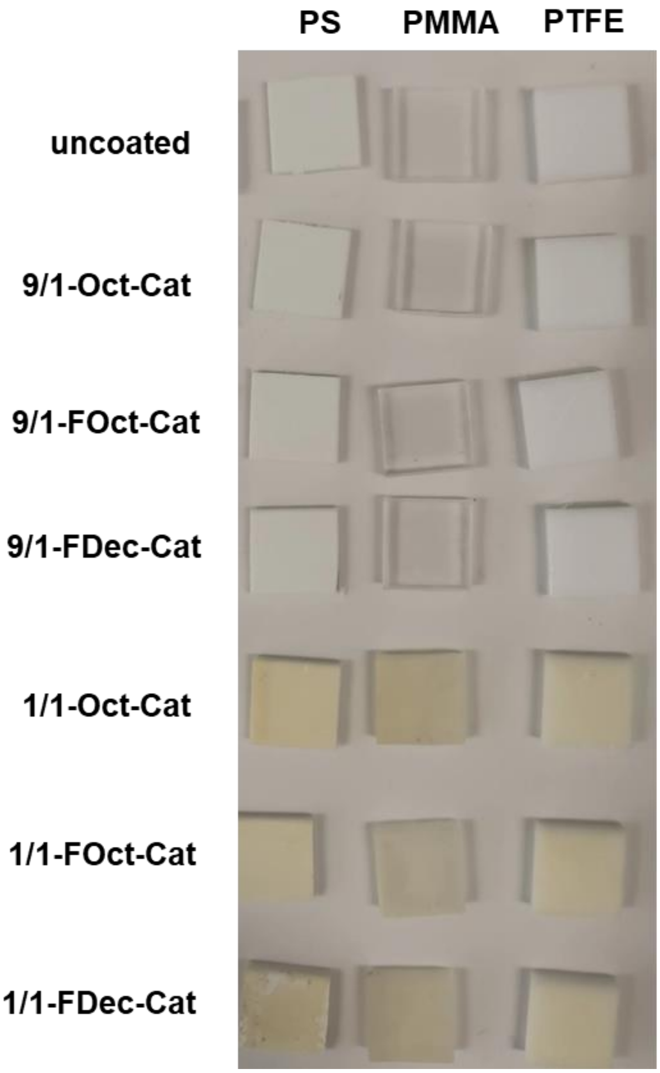

Figure S25. Polymeric surfaces before and after the coating.

Dissipation Plots

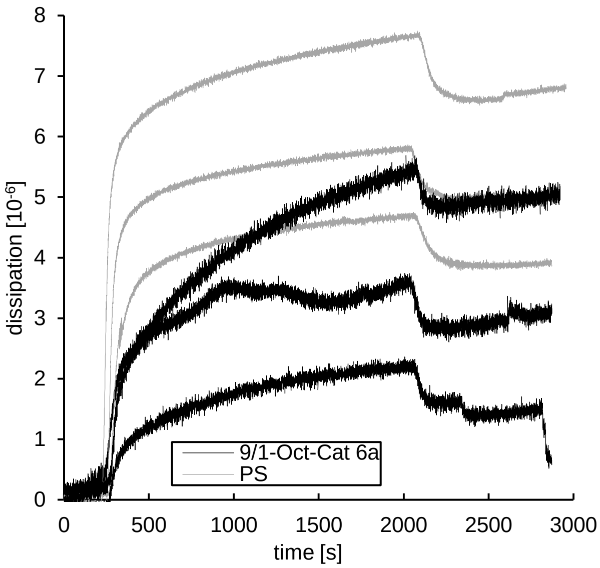

Figure S26. Dissipation change of the third overtones during the adsorption of fibrinogen onto PS-coated sensors (n = 3) without further coating and with an additional coating with 9/1-Oct-Cat **6a**.

Pictures of Coatings 2

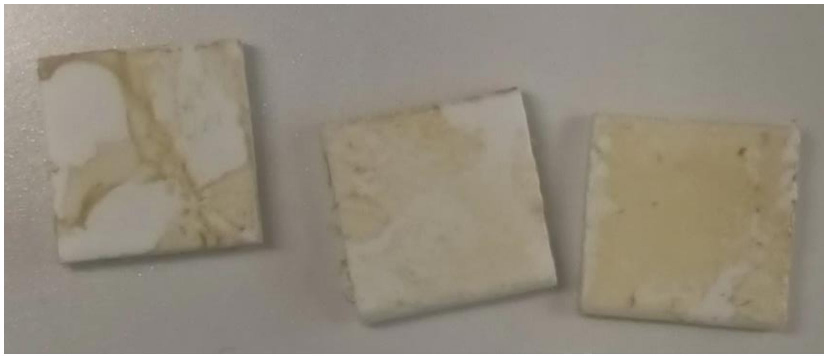

Figure S27. Partial delamination of the 1/1-FDec-Cat **5c** coating on PS.

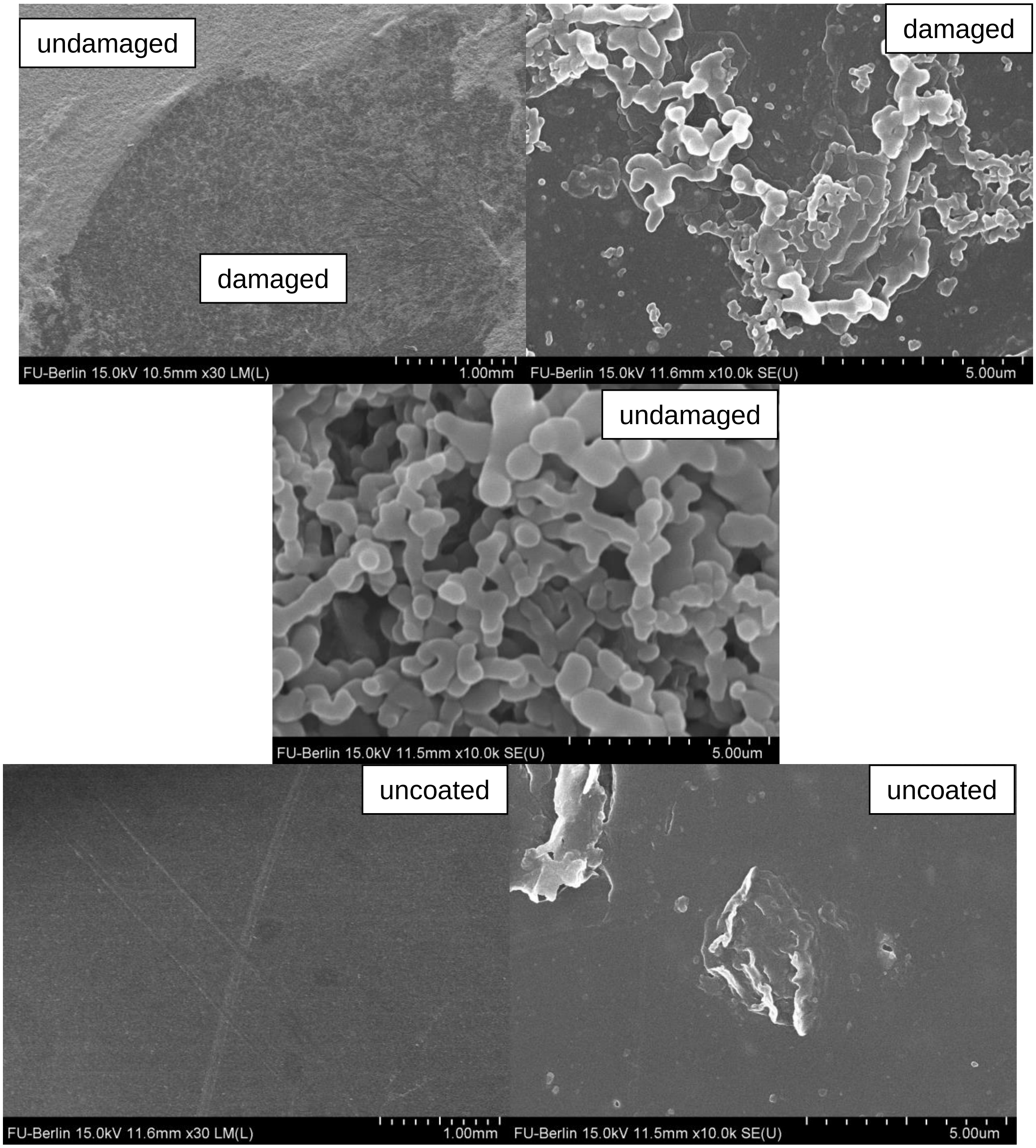

Figure S28. SEM images of damaged vs. undamaged spots on 1/1-FDec-Cat **5c** coating on PS (top & center) and uncoated PS (bottom) as comparison.

Distributions of Hydrodynamic Diameters from DLS

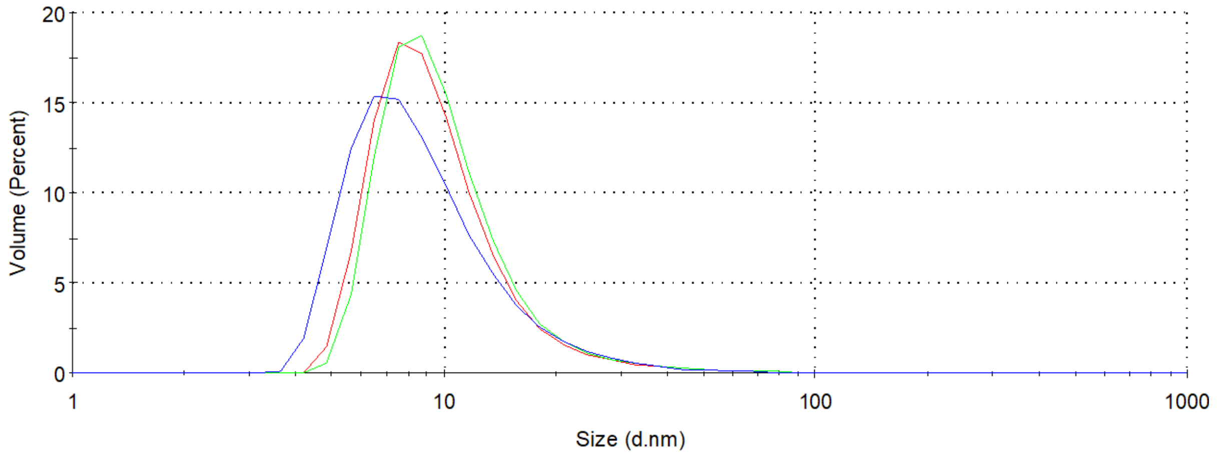

Figure S29. Volume-weighted frequency curves of a solution of 9/1-Oct-Cat **6a** in methanol/water 1/1 (c = 1 mg mL^{-1}).

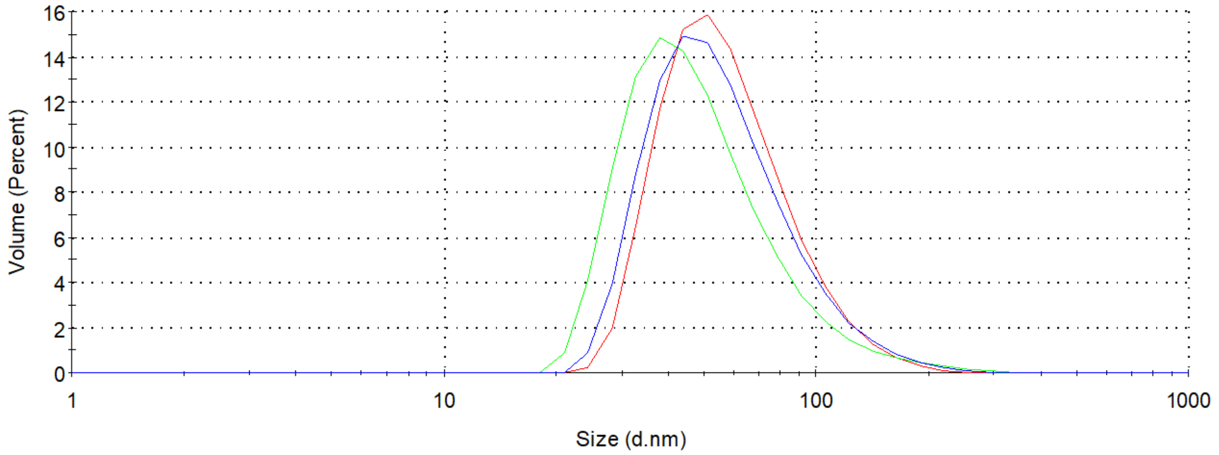

Figure S30. Volume-weighted frequency curves of a solution of 1/1-FOct-Cat **5b** in methanol/water 1/1 (c = 1 mg mL^{-1}).

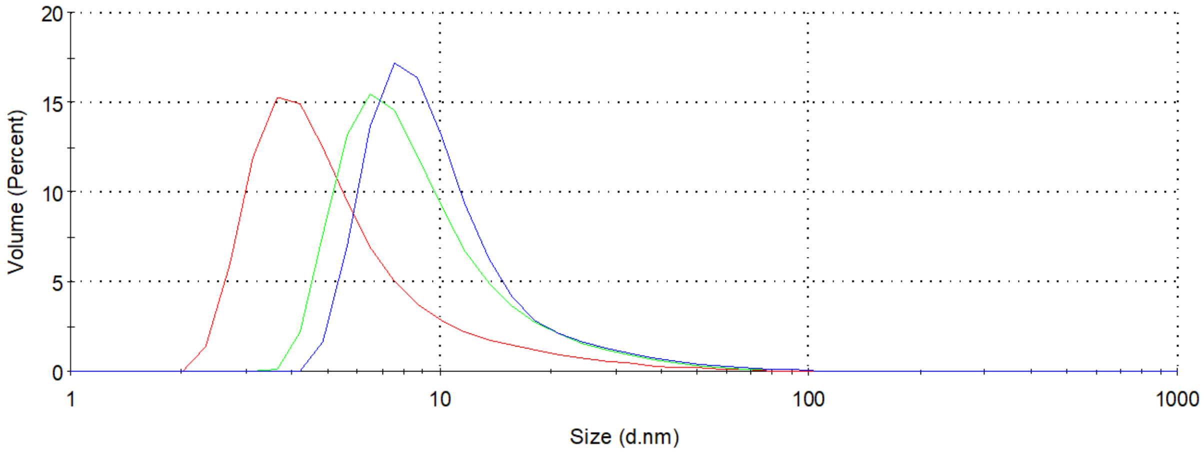

Figure S31. Volume-weighted frequency curves of a solution of 9/1-Oct-Cat **6a** in methanol (c = 1 mg mL^{-1}).

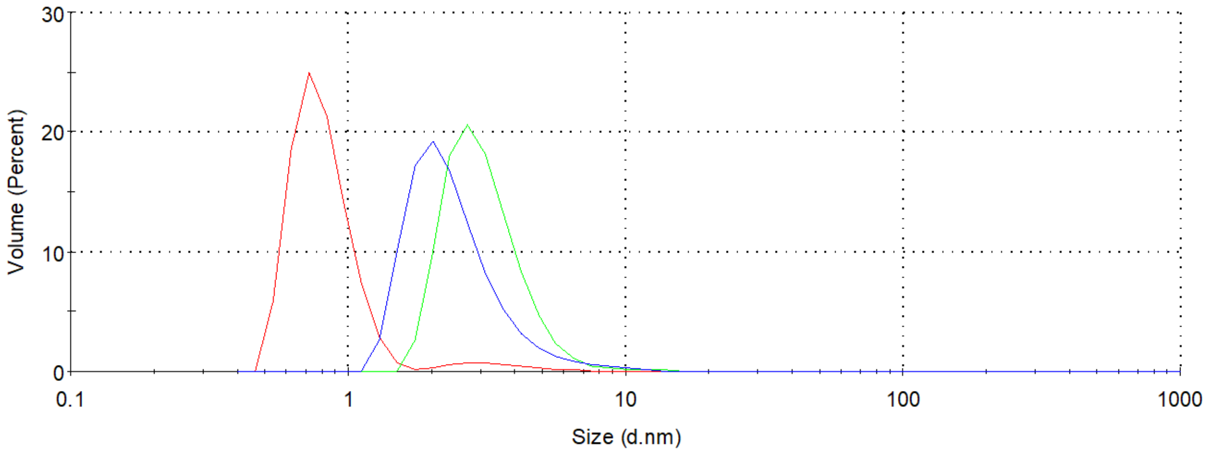

Figure S32. Volume-weighted frequency curves of a solution of 1/1-FOct-Cat **5b** in methanol (c = 1 mg mL^{-1}) (no aggregates).

SEM Images

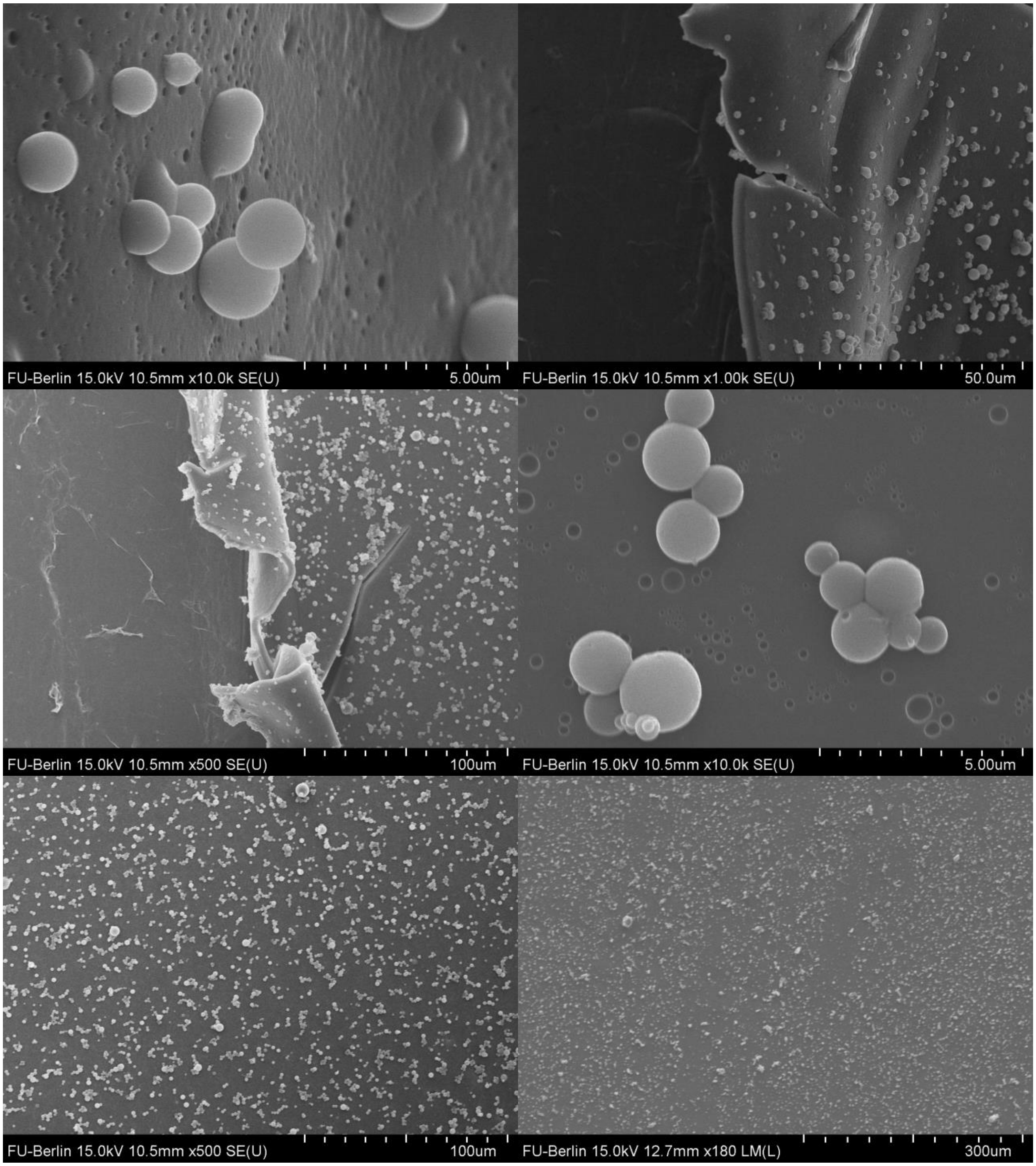

Figure S33. SEM images of a coating of 1/1-FOct-Cat **5b** on PTFE.

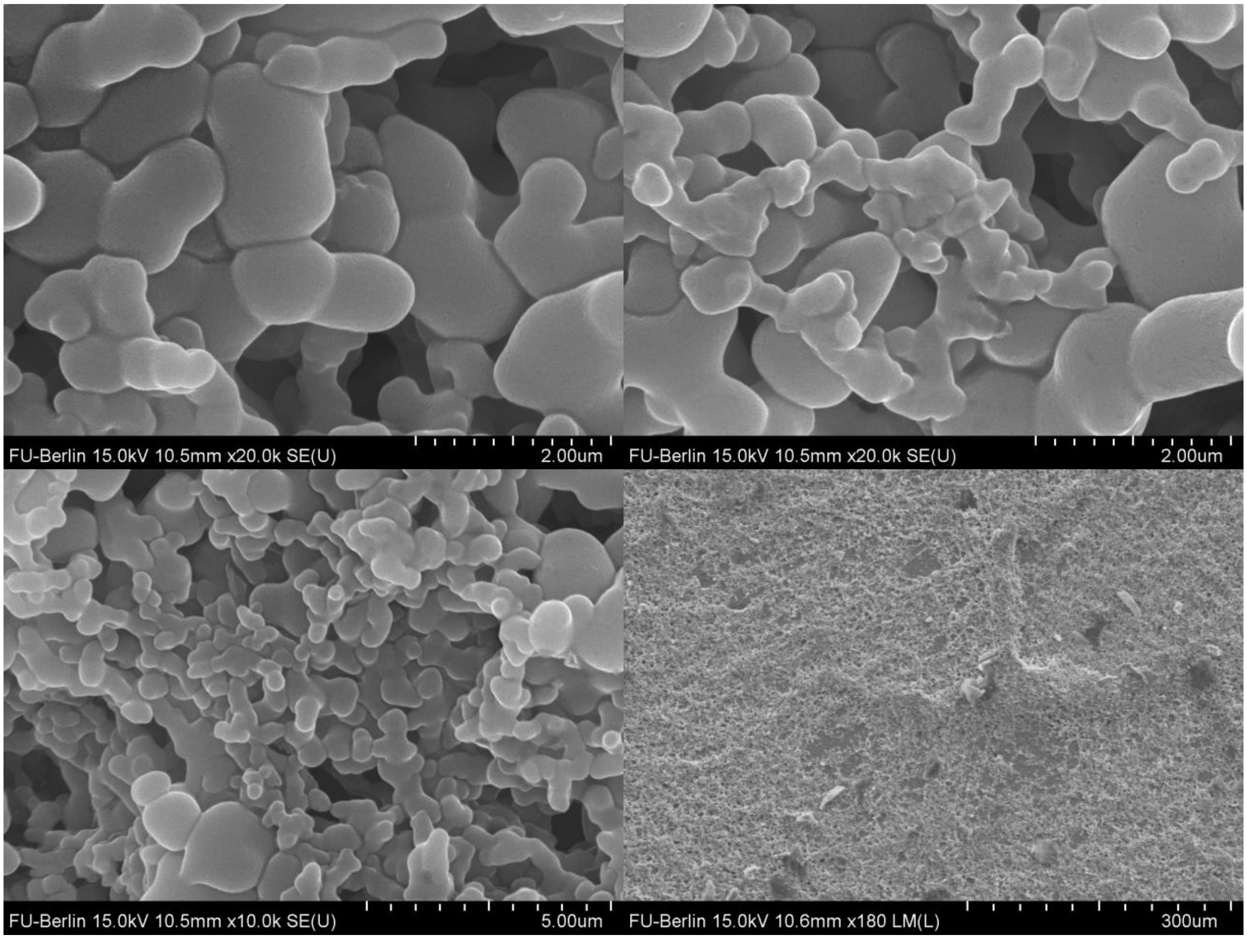

Figure S34. SEM images of a coating of 1/1-FOct-Cat **5b** on PS.

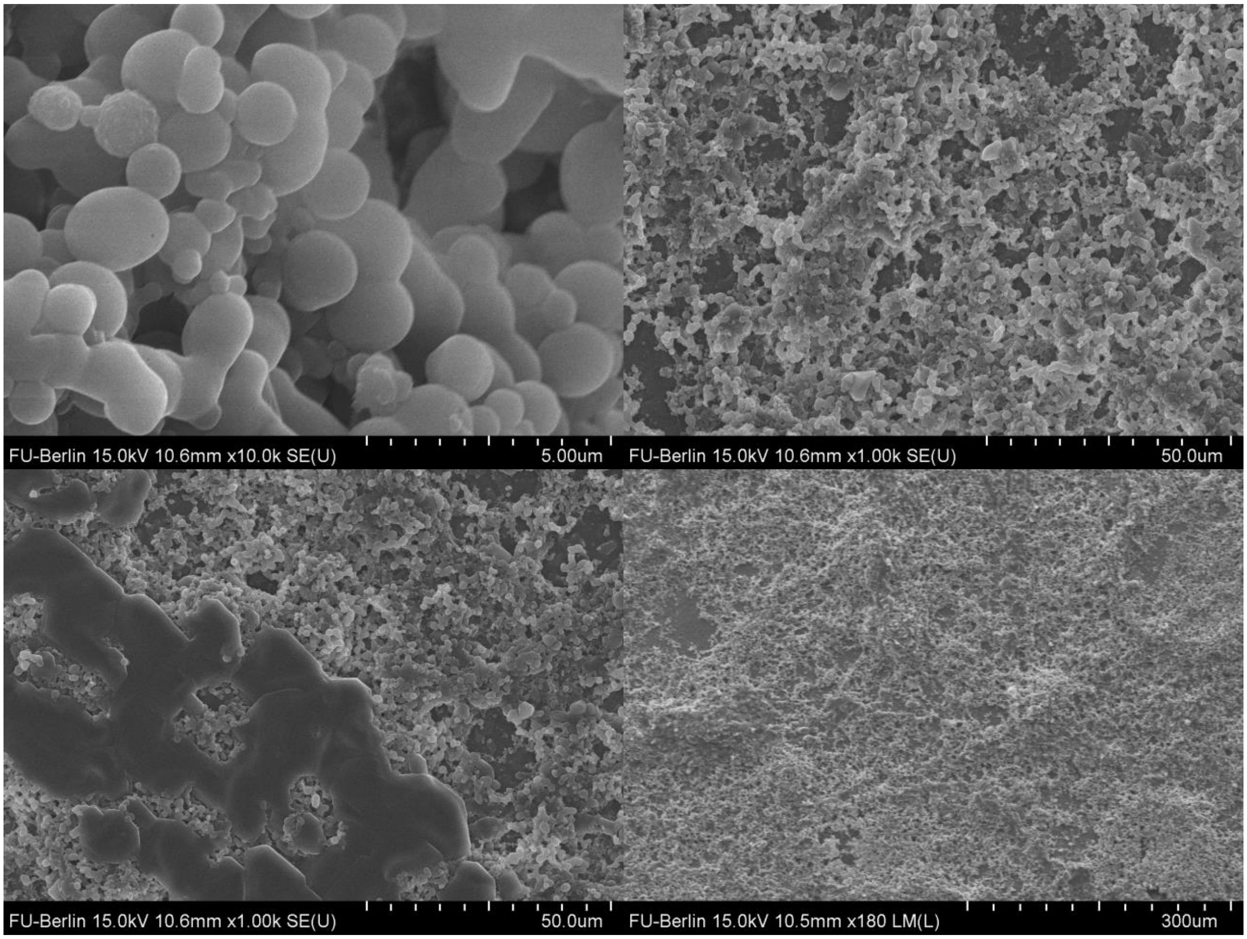

Figure S35. SEM images of a coating of 1/1-FDec-Cat **5c** on PTFE.

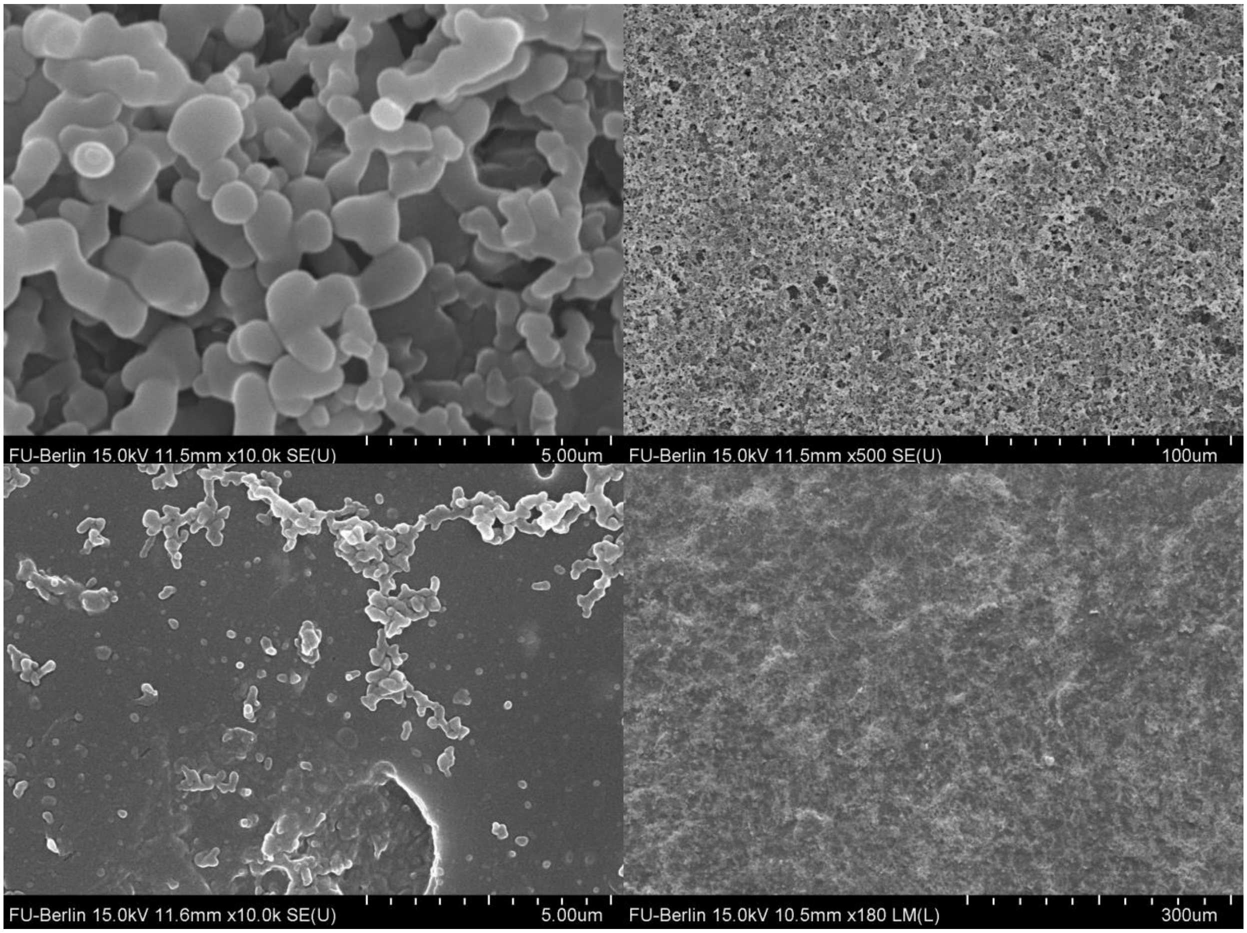

Figure S36. SEM images of a coating of 1/1-FDec-Cat **5c** on PS.

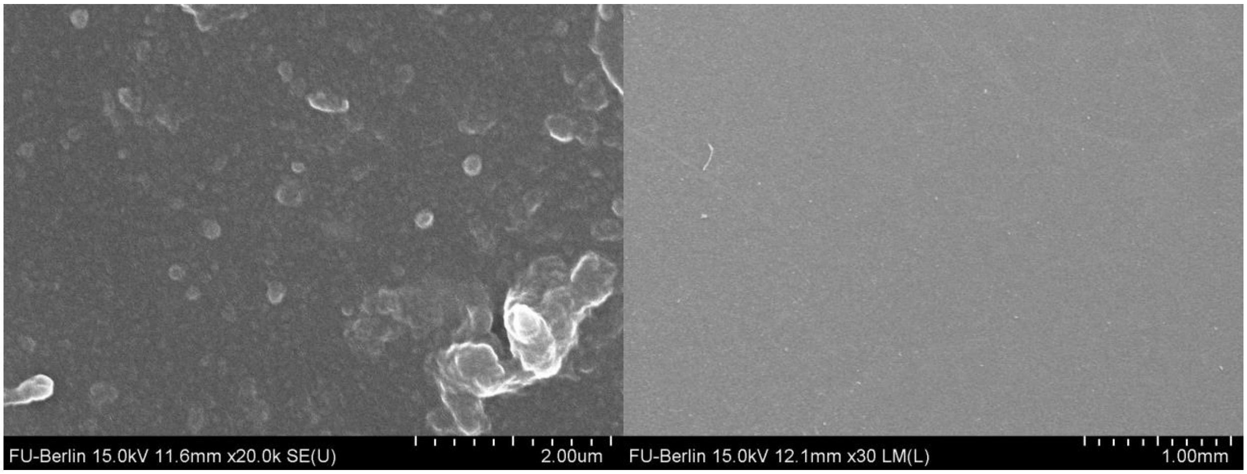

Figure S37. SEM images of a coating of 9/1-Oct-Cat **6a** on PTFE.

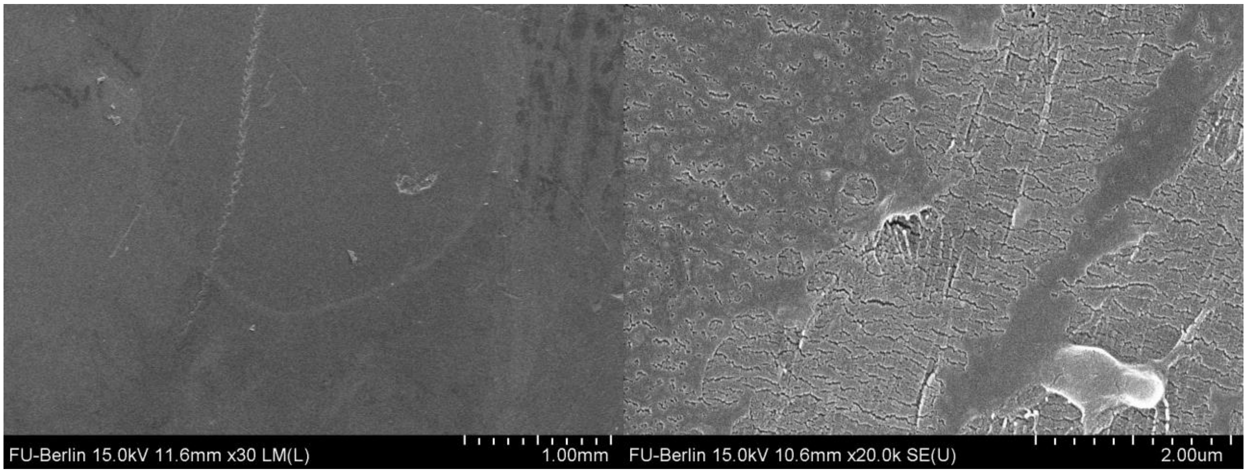

Figure S38. SEM images of a coating of 9/1-Oct-Cat **6a** on PS.

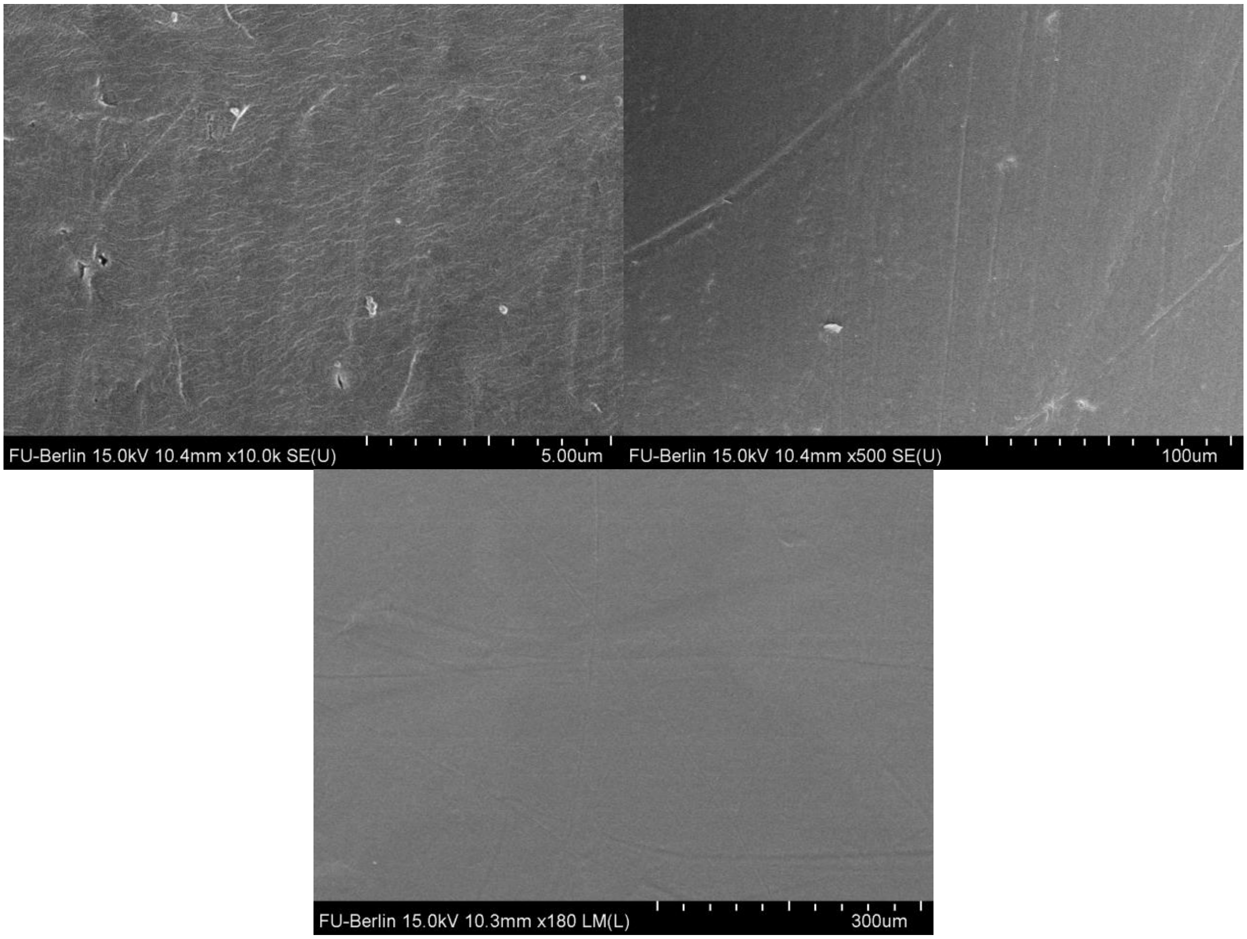

Figure S39. SEM images of uncoated PTFE.

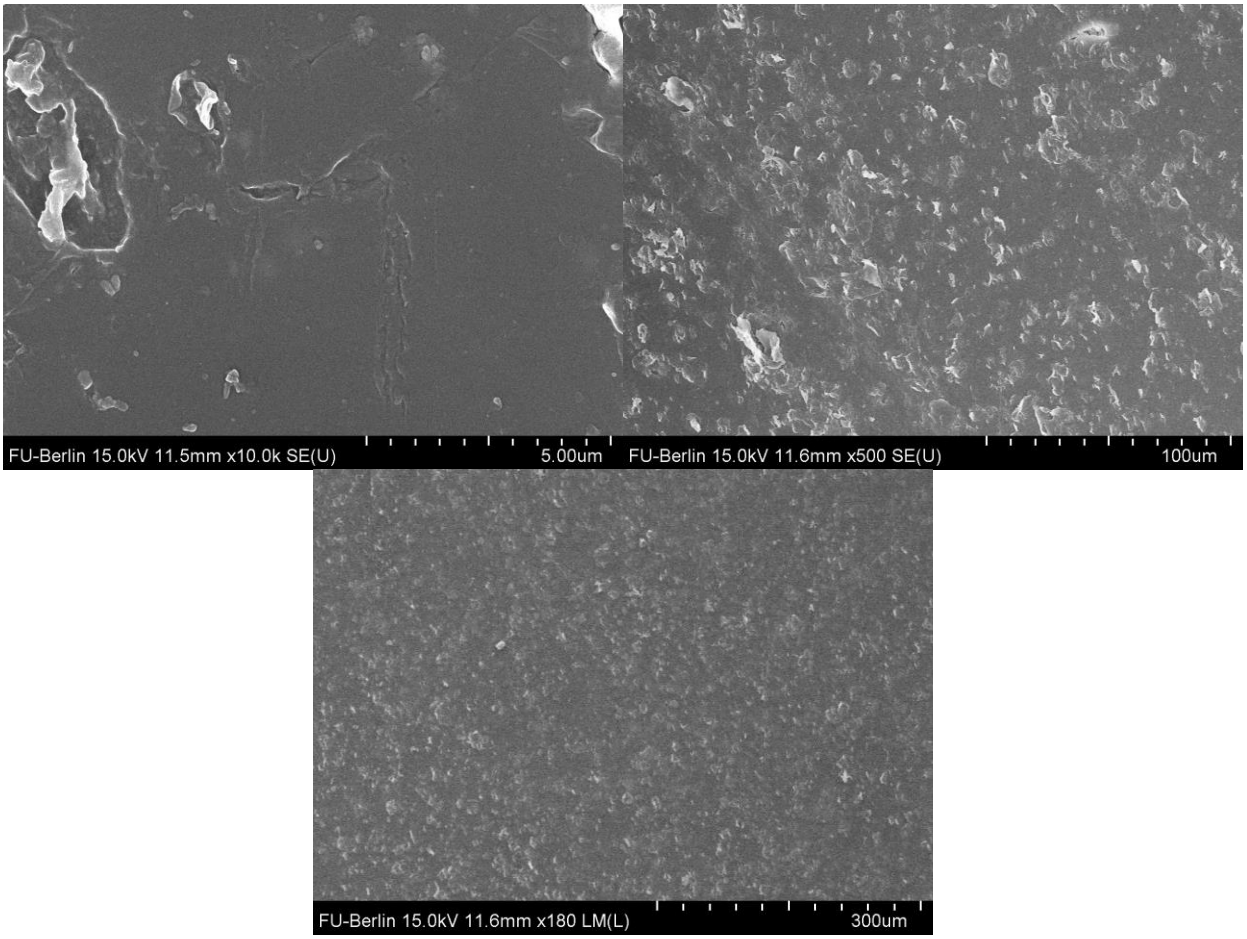

Figure S40. SEM images of uncoated PS.

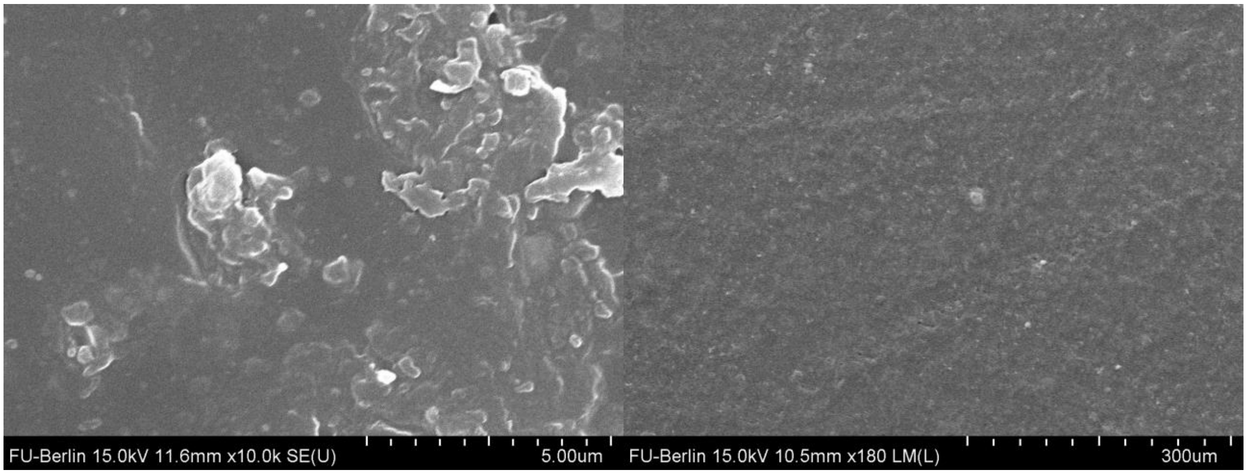

Figure S41. SEM images of monolayers of 1/1-FOct-Cat **5b** on PS.

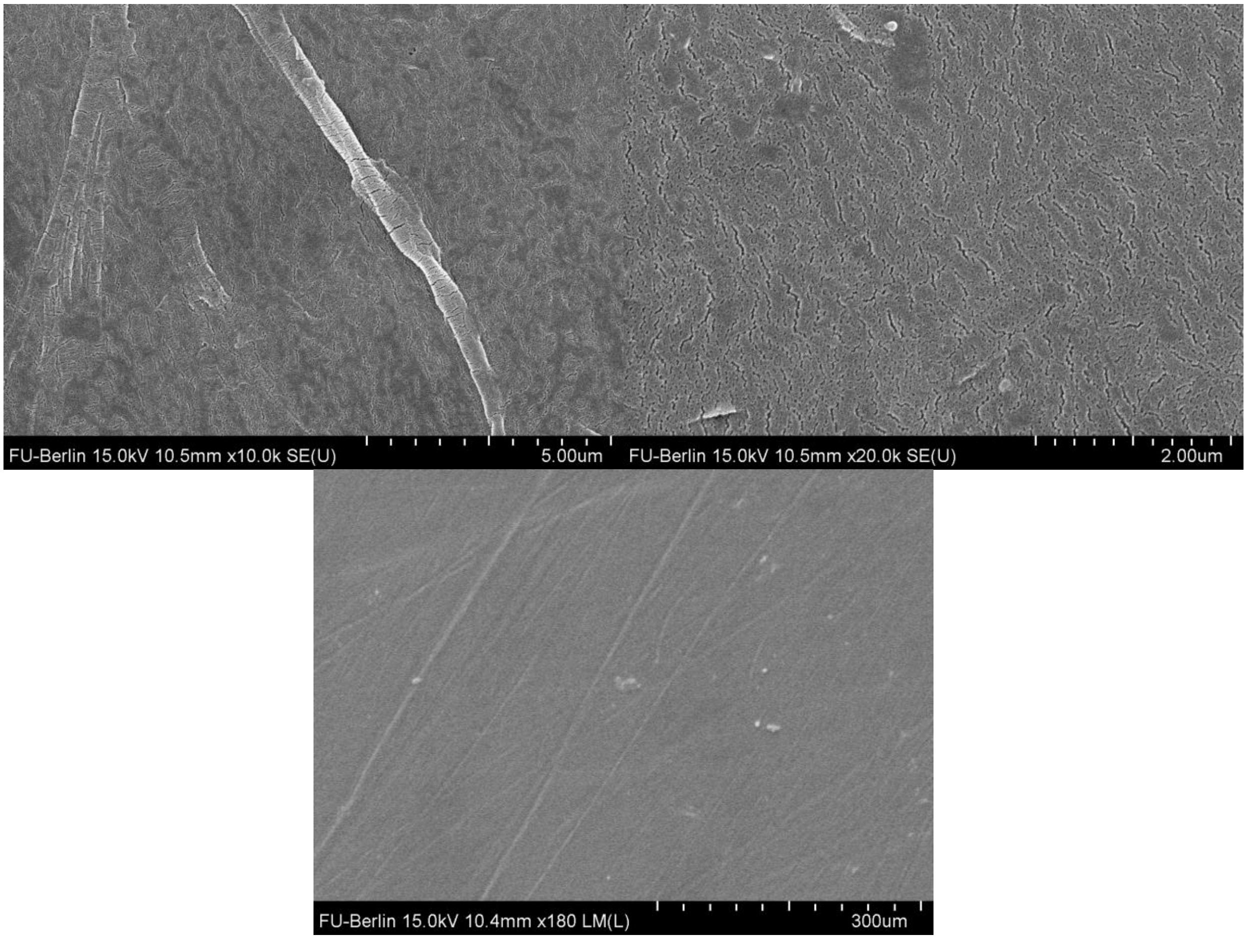

Figure S42. SEM images of monolayers of 1/1-FOct-Cat **5b** on PTFE.
